# Alcohol, cardiovascular disease and industry funding: A co-authorship network analysis of systematic reviews

**DOI:** 10.1016/j.socscimed.2021.114450

**Published:** 2021-11

**Authors:** Su Golder, Jim McCambridge

**Affiliations:** Department of Health Sciences, University of York, York, United Kingdom

**Keywords:** Alcohol drinking, Cardiovascular diseases, Research support, Systematic reviews, Conflict of interest, Alcohol industry, Authorship, Bibliometrics

## Abstract

**Background:**

Alcohol's effects on heart health is the site of a major scientific controversy. We conducted a co-authorship network analysis of systematic reviews on the impacts on alcohol on cardiovascular disease (CVD) in order to investigate patterns of co-authorship in the literature, with particular attention given to industry funding.

**Methods:**

We used Epistemonikos to identify systematic reviews. Review characteristics, influential authors, co-authorship subnetworks, prior histories of alcohol industry funding, study outcomes and citations were investigated.

**Results:**

60 systematic reviews with 231 unique authors met our inclusion criteria. 14 systematic reviews were undertaken by authors with histories of alcohol industry funding, including 5 that were funded directly by the alcohol industry itself. All 14 such reviews identified a cardioprotective effect of alcohol. These formed distinct co-authorship subnetworks within the literature. Of reviews by authors with no prior histories of alcohol industry funding, the findings were mixed, with 54% (25/46) concluding there was evidence of health protective effects. These two groups of reviews differed in other respects. Those with industry funding were more likely to study broader outcomes such as ‘cardiovascular disease’ or ‘coronary heart disease’ as opposed to specific CVD issues such as hypertension or stroke (93% [13/14] versus 41% [19/46]) (chi-squared 12.4, p < 0.001) and have more included studies (mean of 29 versus 20). They were also more widely cited by others. Over time the proportions of systematic reviews on CVD and alcohol undertaken by authors with no prior histories of alcohol industry funding has increased.

**Conclusions:**

Systematic reviews undertaken by authors with histories of alcohol industry funding were more likely to study broader outcomes, and be cited more widely, and exclusively reported favorable conclusions.

## Highlights

• Reviews by authors with no prior industry funding yield mixed findings on alcohol and CVD.
• Reviews by authors with prior industry funding all report alcohol protective effects.
• 14/60 systematic reviews are from industry associated co-authorship subnetworks.
• Reviews by authors with previous industry funding study broader CVD outcomes.
• The latter group of reviews are much more heavily cited.

## 1 Background

Alcohol is well established as a major cause of global burden of disease, with risk increasing with consumption (Griswold et al., 2018). Even though the overall effects of alcohol on health are overwhelmingly negative, since 1974 (Klatsky et al., 1974) a major scientific controversy has emerged about whether small amounts of alcohol may be cardioprotective, as abstainers have worse health than very low level consumers (Oppenheimer and Bayer, 2020). Much attention has centred on the limits of observational epidemiology (Holmes et al., 2014), and how far abstainers include both ‘sick quitters’ and those likely to have worse outcomes for other reasons (Shaper et al., 1988). There is substantial unexplained heterogeneity in meta-analytic studies (Roerecke and Rehm, 2012). Alcohol consumption is challenging to measure well, likely biasing towards the null (Naimi et al., 2013). Possible cardiovascular disease (CVD) effects belong to a wider set of claimed health benefits of alcohol, many of which have no plausible biological mechanisms, nor obvious relationships to each other (Fekjaer, 2013). The controversies remain unresolved today.

The alcohol industry have sponsored studies in this literature (McCambridge and Hartwell, 2015), and use evidence of purported health benefits in seeking to influence public policy (McCambridge et al., 2018; Savell et al., 2016). It is well known that other powerful corporate sectors have sponsored and shaped science deliberately to distract from the damage caused by their activities (Oreskes and Conway, 2010; McGarity and Wagner, 2012; Popp JB et al., 2018; Bes-Rastrollo et al., 2013; Mandrioli et al., 2016; Dunn et al., 2014; Hansen et al., 2019; Newman, 2004). We do not know whether the alcohol industry has behaved like the tobacco industry in perpetrating a decades-long conspiracy to subvert the peer-reviewed science base (Proctor, 2012). The first quantitative study (McCambridge and Hartwell, 2015) found no evidence that alcohol industry funding biased what is known about possible cardioprotective effects of alcohol in meta analytic data, apart from with regard to stroke; this was a preliminary study, however, and used a crude measure of concern about industry funding (McCambridge and Hartwell, 2015). The second quantitative study was undertaken by the alcohol industry; this found no associations between alcohol industry funding and a range of health outcomes, including CVD, in meta analytic studies (Vos et al., 2020). Major alcohol companies recently funded the first clinical trial in this area, which was then stopped because the biased nature of the study (for example, not adequately studying negative outcomes) was identified soon after the trial began (Mitchell et al., 2020). After the researchers approached them, the alcohol companies agreed to fund the Moderate Alcohol and Cardiovascular Health (MACH) trial to advance their commercial interests (Mitchell et al., 2020). The MACH trial shows that investigations of the mechanisms by which industry funding may bias science must give attention to the conduct of researchers.

Systematic reviews are important because decisions in public health rarely get made on the basis of individual studies (Centre for Reviews and Di, 2009). Collaborations in research, including co-authorship of reviews, is increasingly encouraged by developments in funding and wider trends in science.(Koseoglu, 2016; Carpenter et al., 2014) Social network analysis (Fonseca et al., 2016) is one method that can be applied to study relationships between authors, capable of examining relationship structure and connections between people, formation of groups or cliques, and identifying core actors or influencers in co-authorship networks.

Given that there are a large number of existing systematic reviews on alcohol and CVD we decided to study co-authorship patterns in reviews using a network analysis approach. This could help identify collaboration trends, leading researchers, clusters of authors and “invisible communities” as networks.(Popp JB et al., 2018; Fonseca et al., 2016) This approach is well suited to uncovering scientific collaborations amongst review authors and connections to alcohol industry funding sources, including both alcohol companies and related organisations. This study therefore seeks to investigate patterns of co-authorship in the reveiw literature, with particular attention given to network structures and histories of industry funding.

## 2 Methods

We carried out a co-authorship network analysis in which we identified individual authors of systematic reviews and the relationships between them, that is, whether they have co-authored reviews together. We followed four main steps to undertake our co-authorship analysis. Firstly, we retrieved systematic reviews focusing on the impact of alcohol on CVD. Secondly, we extracted data from each of the included systematic reviews (such as publication year, number of authors). Thirdly, for each author we recorded any known previously declared connections to the alcohol industry (see below). Note, this refers to any history of alcohol industry funding and thus largely does not indicate ongoing funding. Lastly, we carried out our analysis which included a visualisation of our network of authors, metrics which help to identify the most influential authors in the network and an analysis of the characteristics of the systematic reviews (Fonseca et al., 2016). Data collection and analysis was undertaken by the first author. The second author supervised the study, checked all data and categorized outcomes blind.

### 2.1 Phase 1: retrieval of systematic reviews focusing the impact of alcohol on CVD

#### 2.1.1 Search strategy

We searched for systematic reviews in Epistemonikos on the May 6, 2020 via https://www.epistemonikos.org/using the following search strategy;

(alcohol* OR drinkers OR drinking OR beer OR wine OR spirits).

We selected Epistemonikos as it is the most comprehensive freely available source of systematic reviews and is populated by regularly searching ten databases including PubMed, EMBASE and the Cochrane Database of Systematic Reviews (CDSR). This search retrieved 1844 records which were related to many aspects of health. In order to identify those records related to solely to CVD we entered these records into an Endnote Library and conducted a search with the following terms in any field;

(Cvd OR Cardio* OR chd OR heart OR cardiac OR coronary OR myocard* OR angina OR ischemic attack OR ischaemic attack OR peripheral atrial OR aortic disease OR aortic aneurysm OR ventricular dysfunction OR mortality OR stroke OR intracerebral hemorrhage OR cerebrovascular accident OR blood pressure OR hypertension).

We included the search term ‘mortality’ as systematic reviews that examined ‘all-cause mortality’ were likely to include studies of cardiovascular impacts. We conducted the search in these two stages because of the limited search interface provided by Epistemonikos.

#### 2.1.2 Eligibility criteria

To meet our inclusion criteria studies were required to be a systematic review (with or without a meta-analysis). Eligible systematic reviews were required to have studied any adult population with a focus on alcohol intake (as the exposure), a comparator of no alcohol or lower alcohol intake, and any CVD as the primary outcome. We did not apply date restrictions, and due to logistical constraints we did not include reports in languages other than English.

#### 2.1.3 Selection of studies

The titles and abstracts were then sifted by two researchers independently and the full-texts of all potentially relevant articles examined so that we could assess if they met all our inclusion criteria. Any disagreements were resolved by discussion.

### 2.2 Phase 2: data extraction from systematic reviews

For each systematic review we extracted data on publication year, journal title, number of authors, countries of institutional affiliations, declared funding sources (for the study itself and the authors conflict of interest disclosures), number of references, number of included studies, main CVD conditions studied and number of times the review is cited (Web of Science, Core Collection, Searched November 28, 2020). The main CVD conditions studied were important to extract given the many possible different disease categories such as coronary heart disease, stroke, and heart failure.

Two reviewers (the authors) independently assessed whether the conclusions indicated that evidence was provided for or against any protective effect of low dose alcohol consumption. The latter reviewer was blinded to any author, journal or funding information and made their assessment based on the stated conclusions (and in some cases additional results text) only.

### 2.3 Phase 3: standardisation of entries for authors and identification of alcohol industry funding

Cleaning the data involved checking for inconsistencies in names, authors with the same name and typographical errors. For each unique author we then identified any known history of alcohol industry funding. We were able to assess whether funding had been previously declared by the authors by conducting a search of the Organization-Enhanced [Index], Organization, Suborganization, Funding Agency, and Funding Text fields for known alcohol companies and related organisations in the Web of Science suite of database using a search strategy reported elsewhere (Golder, 2020). In addition to the funding sections of journal article acknowledgements, declarations of interest are processed in Web of Science.

We defined any prior direct financial support to the author to undertake research from alcohol companies or related organisations to constitute a history of alcohol-industry funding and used this definition in our analysis. In addition, we made a note of any other support to the author, for instance, for attending conferences or positions on alcohol industry sponsored scientific committees and instances of co-authors in receipt of alcohol-industry funding (Supplementary Table 1). Although we did not include this information in our definition of industry research funding we note that there were only two authors of the systematic reviews that did not have alcohol industry research funding who received financial support to attend scientific meetings (Supplementary Table 1).

### 2.4 Phase 4: network visualisation and analysis

In order to construct and visualise the co-authorship network we used the open source software Gephi: https://gephi.org/. Each unique author is represented by a circular shape, the size of the circle depicts the number of systematic reviews published by that author. Any line connecting a pair of authors represents co-authorship, and the thickness of the line is weighted by the number of publications co-authored by that pair of authors. The network graph allowed us to visualise groups of authors connected directly (co-authorship on the same paper) and indirectly (connected through a mutual co-author on separate papers).

To uncover the most influential researchers the following methods were used; productivity of the authors (number of systematic reviews authored), number of co-authorships (degree centrality), prominence of the author's position in the network (i.e. how much an author connects other authors via the shortest path possible - betweeness centrality) and how close an author is connected to all other authors (closeness centrality). In addition, we looked for link authors. These are authors who connect two subnetworks together. The removal of link authors would result in two or more separate subnetworks, so we consider the link authors to be influential connectors who help bind the network together.

We examined trends in research patterns over time. For example, the size of the overall network and each sub-network was measured using the number of authors and number of co-authorships. The density of the subnetwork was calculated by dividing the number of co-authorships that exist with the maximum possible number of co-authorships that can exist. The higher the density, therefore, the more authors are connected to each other.

We compared reviews with and without authors with any known alcohol industry research funding histories using the following metrics; health conditions studied, type of journal published in, number of authors, productivity of authors, number of references, number of included studies and number of times the review is cited in the Web of Science core collection.

## 3 Results

The CVD search in endnote of the 1844 records from Epistemonikos yielded 270 records. After title and abstract screening, full texts were examined for 91 potentially relevant systematic reviews, with 31 excluded. Eight were not systematic reviews (Rotondo et al., 2001; Hansel et al., 2012; Sinkiewicz et al., 2014; Arredondo Bruce and Del Risco Morales, 2014; de Gaetano et al., 2003; Leino et al., 1998; Djousse and Gaziano, 2008; McKee and Britton, 1998), seven were either letters, editorials, meeting abstracts or summary paper of an included study (Costanzo et al., 2011a; Lin et al., 2010; Liu et al., 2013; Roerecke and Rehm, 2013; Mostofsky et al., 2016a; Cho et al., 2017; de Gaetano et al., 2002) six were systematic reviews in which alcohol was not evaluated as risk factor for CVD (McCambridge and Hartwell, 2015; Cheng et al., 2019; Lee et al., 2019; Raheja et al., 2018; Romanowicz et al., 2011; Richard et al., 2013), five were in a language other than English (VillarinoMarín et al., 2002; Liu et al., 2010a, 2010b; Wang et al., 2008; Chemello et al., 2010), three were not focused on CVD (Corrao et al., 1999, 2004; Wang et al., 2014) and two were methodology papers (Supplementary Figure 1). (Klatsky and Tran, 2016; Wallach et al., 2020) Sixty systematic reviews (Roerecke and Rehm, 2012; Bagnardi et al., 2008; Barbalho et al., 2010; Briasoulis et al., 2012; Brien et al., 2011; Britton and McKee, 2000; Chen et al., 2008; Cleophas, 1999; Corrao et al., 2000; Costanzo et al., 2010; Costanzo et al., 2011b; Di Castelnuovo et al., 2006; Di Castelnuovo et al., 2002; Drogan et al., 2012; Gallagher et al., 2017; Green et al., 2013; Huang et al., 2014; Huang et al., 2017; Jung et al., 2019; Karpyak et al., 2014; Kelso et al., 2015; Kodama et al., 2011; Koppes et al., 2006; Larsson et al., 2014; Larsson et al., 2015; Larsson et al., 2018; Larsson et al., 2016; Lippi et al., 2015; Mazzaglia et al., 2001; McFadden et al., 2005; Mostofsky et al., 2016b; Naame et al., 2019; O'Neill et al., 2018; Padilla et al., 2010; Patra et al., 2010; Peng et al., 2020; Rehm et al., 2017; Reynolds et al., 2003; Rimm et al., 1996; Rimm et al., 1999; Roerecke et al., 2013; Roerecke et al., 2017; Roerecke and Rehm, 2010; Roerecke and Rehm, 2011; Roerecke and Rehm, 2014a; Roerecke and Rehm, 2014b; Roerecke et al., 2018; Ronksley et al., 2011; Samokhvalov et al., 2010; Spencer et al., 2017; Stockwell et al., 2016; Taylor et al., 2009; Xin et al., 2001; Yang et al., 2016; Ye et al., 2019; Yoon et al., 2020; Zhang et al., 2014; Zhang et al., 2015; Zhao et al., 2017; Zheng et al., 2015) remained (Supplementary Table 2).

### 3.1 Characteristics of the systematic reviews

The 60 systematic reviews were published from 1996 to 2020, with the majority (45 reviews) published after 2010. The reviews were published in a wide range of journal titles with the most common being ‘Addiction’ and the ‘BMJ’ (4 reviews each). There were 231 unique authors of the 60 systematic reviews with the number of authors per systematic review varying from one to 13 (mean 5). The 231 authors were affiliated to institutions in 18 different countries.

### 3.2 Influential authors

The most influential authors within our network of the 231 review authors were identified using a variety of measures (Table 1). Five authors were link authors (that is they connected two or more sub-groupings within subnetworks, and without them the subnetworks would be separated); Britton, Bagnardi, Rimm, Mukamal and He Jiang. Rehm collaborated with the highest number of authors (18) followed by Roerecke (15) (degree centrality, Table 1). The four most common co-authors between other authors were Rehm, Rimm, Bagnardi and Mukamal (betweenness centrality). When we looked at how close a particular author is connected to other authors in their subnetwork many of the authors scored one or close to it (closeness centrality).

**Table 1:** Top Ten Influential authors.

Author	Country affiliation	Link author	Betweenness centrality^a^	Closeness centrality^a^	Degree centrality^a^	No. of systematic reviews authored	Alcohol industry funding history
j. rehm (subnetwork 1)	Canada, Germany, Switzerland	No	60.8	1	18	12	None known
e. b. rimm (subnetwork 3)	USA, Canada	Yes	51	0.777778	10	3	International Life Sciences Institute (ILSI Europe Alcohol Task Force)
v. bagnardi (subnetwork 2)	Italy	Yes	46.5	0.928571	12	3	None known
k. j. mukamal (subnetwork 3)	USA	Yes	40	0.7	8	3	Anheuser-Busch InBev, Carlsberg Breweries A/S, Diageo plc, Heineken, Pernod Ricard USA LLC
m. roerecke (subnetwork 1)	Canada	No	27.3	0.857143	15	10	None known
a. britton (subnetwork 5)	England	Yes	27	1	10	3	None known
he jiang (subnetwork 8)	USA	Yes	25	1	10	2	None known
e. s. shin (subnetwork 7)	Korea	No	12	1	11	2	None known
j. g. jung (subnetwork 7)	Korea	No	12	1	11	2	None known
o. s. m. hasan (subnetwork 1)	Canada	No	8.8	0.72	11	3	None known

a Betweenness centrality measures the number of times an author acts as a bridge along the shortest path between two other authors, closeness centrality is based on the ‘closeness’ of authors to other authors, and degree centrality is based on the number of connections held by each author. The table is ranked according to betweenness centrality as this has the added advantage that a fully connected graph is not required whereas closeness centrality is measured within the relevant subnetwork.

### 3.3 Subnetworks within the overall network

We identified 31 subnetworks in our network, including 22 reviews where the authors were not involved in any other review, so these formed 22 of the 31 subnetworks. The other nine subnetworks authored the remaining 38 systematic reviews (Table 2).

**Table 2:** Summary characteristics of systematic reviews in each subnetwork in the network.

Subnetwork	No of systematic reviews	No of unique Authors	Mean no of authors per review	Years Published	Mean no of times systematic reviews cited	Country affiliations of authors	Funding sources for systematic review	Other alcohol industry funding to authors	Health conditions studied
Subnetwork 1	12	20	4 (2–9)	2009–2018	101 (23–195)	Canada, Australia, Germany, Spain, Switzerland	Public funding and pharmaceutical company	None identified	Hypertension, stroke, coronary heart disease, atrial fibrillation, blood pressure, cardiovascular disease
Subnetwork 2	6	14	5 (5–6)	2000–2011	336 (122–615)	Italy, Finland, Poland	Public funding and alcohol industry-related organisations – ERAB, Cervisia Consulenze	**Costanzo:** ERAB, Assobirra, Cervisia Consulenze, International Organization of Vine and Wine (OIV) **Di Castelnuovo, Iacaviello, de Donati,** Cervisia Consulenze, ERAB **Gaetano** Cervisia Consulenze, ERAB, Assobirra **La Vecchia:** Assobirra **Zambon**: ERAB	Cardiovascular disease, coronary heart disease, vascular risk
Subnetwork 3	5	15	5 (4–5)	1996–2016	529 (62–927)	Canada, USA, France, Netherlands	Public Funding, University, and Alcohol Industry-Related Organisations International Life Sciences Institute (ILSI Europe Alcohol Task Force)	**Criqui, Fosher, Grobbee, Rimm, Stampfer, and Williams:** ILSI Europe **Klatsky:** ABMRF, ILSI Europe **Mukamal:** Foundation for NIH from the alcoholic beverage industry (Anheuser-Busch InBev, Carlsberg Breweries A/S, Diageo plc, Heineken, Pernod Ricard USA LLC)	Cardiovascular disease, coronary heart disease
Subnetwork 4	4	6	3 (3–4)	2014–2018	68 (10–154)	Sweden	Public funding and university	None identified	Heart failure, atrial fibrillation, stroke
Subnetwork 5	3	11	4 (2–7)	2000–2018	79 (13–154)	England, Scotland, France	Public funding and not reported	None identified	Coronary heart disease, stroke
Subnetwork 6	2	7	6 (5–6)	2016–2017	122 (61–182)	Canada, Australia, USA	Public funding	None identified	Cardiovascular disease, coronary heart disease
Subnetwork 7	2	12	7 (6–8)	2019–2020	1 (0–1)	Korea	Public funding	None identified	Cardiovascular disease, hypertension
Subnetwork 8	2	13	8 (7–9)	2014–2015	48 (29–67)	China	Public funding	None identified	Cardiovascular disease, stroke
Subnetwork 9	2	11	6 (6)	2001–2003	501 (445–556)	USA	Public funding	None identified	Blood pressure, stroke
Subnetworks 10 to 31 (Isolated subnetworks -where all authors of a systematic review have only authored that one review)	22	124	6 (1–13)	1999–2020	43 (0–186)	Brazil, Netherlands, Germany, Australia, Scotland, China, USA, Japan, Italy, England	Public funding, university, alcohol industry-related organization (Life Sciences Institute (ILSI Europe Alcohol Task Force) and not reported	**Boeing:** Beer and Health Foundation **Bouter:** ILSI Europe **Dekker**: ILSI Europe, Heineken **di Giuseppe:** ERAB **Djousse:** ABMRF **Heine:** Heineken, ILSI Europe **Hendriks**: ILSI Europe, ERAB, Dutch Foundation for Alcohol Research, Carlsberg **Koppes:** ILSI Europe, Heineken	Cardiovascular disease, coronary heart disease, hypertension, blood pressure, myocardial infarction, atrial fibrillation, abdominal aortic aneurysm, atherosclerosis, heart rate, venous thromboembolism, heart failure, Cerebral Hemorrhage, stroke, coronary artery disease, lipid profile

The largest subnetwork (subnetwork 1) consisted of 12 systematic reviews with 20 authors representing 20% (12/60) of all reviews, and a smaller proportion of authors – 9% (20/231) (Table 2).

### 3.4 Funding sources

Five systematic reviews were funded directly by alcohol industry organisations (Cervisia Consulenze, European Research Advisory Board (ERAB), and International Life Sciences Institute (ILSI) Europe Alcohol Task Force). A further nine systematic reviews had authors who have received industry-related funding for other studies (from Alcoholic Beverage Medical Research Foundation (ABMRF), Assobirra, Beer and Health Foundation, Carlsberg, Cervisia Consulenze, Dutch Foundation for Alcohol Research (SAR), ERAB, European Forum for Responsible Drinking, Heineken, International Organisation of Vine and Wine (OIV) and ILSI Europe Alcohol Task Force). Of the 46 reviews with no known industry funding connections, 34 received public funding (from government or inter-government agencies/organisations), three received funding from universities (one in addition to public funding) and two received funding from pharmaceutical companies (one in addition to public funding). Nine reviews did not report any funding source.

**Fig. 1:**
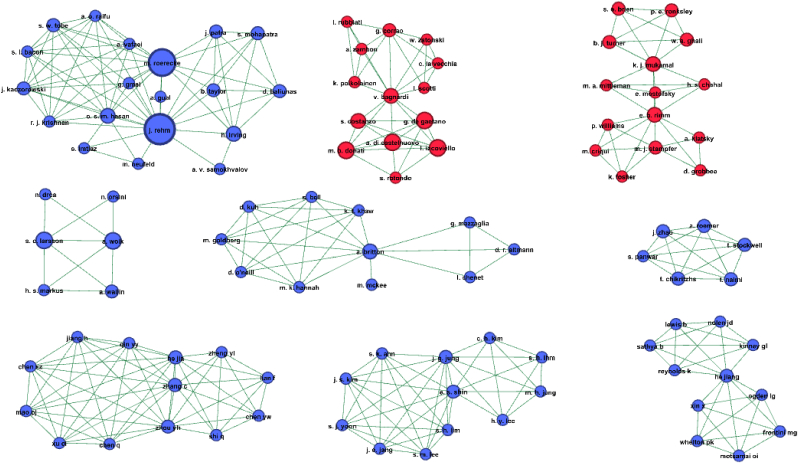
Co-authorship network analysis: Subnetworks 1 to 9. Legend: Colour coding of authors of systematic reviews. Blue (72.9%): author has no known history of alcohol industry research funding. Red (27.1%): author has previously received alcohol industry research funding. (For interpretation of the references to colour in this figure legend, the reader is referred to the Web version of this article.)

Fig. 1 presents subnetworks 1–9 categorising authors by any history of alcohol industry funding. Three of the subnetworks 10–31 (isolate reviews) also had any known alcohol industry funding history (Supplementary Table 1).

### 3.5 Systematic review conclusions and industry funding history

There was high level of agreement in assessment of the review conclusions made by the two reviewers (57 out of 60). Only 3 reviews required discussion (none were funded by the alcohol industry, and all were judged by one reviewer unclear or uncategorizable). 39 reviews concluded there was evidence for some form of CVD health protection effect of alcohol consumption at low doses, mostly in line with a J-shaped (though in some cases U-shaped) risk curve. Two reviews were inconclusive stating that ‘inconsistent results emerged on the j-shaped relationship’ (Mazzaglia et al., 2001) and ‘relationship between low-middle alcohol consumption and ICH remains controversial’ (Peng et al., 2020) respectively. The other 19 reviews (32%) concluded there was no evidence for any protective effect of alcohol consumption. Review findings were strongly related to having any prior history of industry funding - all 14 such reviews concluded that alcohol has CVD health protection effects, whereas the other 46 systematic reviews were quite evenly divided in reaching such conclusions (Table 3). The Fisher's Exact Test Statistic was 10.654 and p = 0.002 revealing that conclusions on cardioprotection significantly differed by alcohol industry funding history.

**Table 3:** Systematic reviews conclusions and alcohol industry funding.

Subnetwork and number of SRs	Concludes alcohol had cardio protective effect	Concludes alcohol does not have any cardio protective effect
Known histories of alcohol Industry funding (all authors)		
Subnetwork 2 (n = 6)	6	0
Subnetwork 3 (n = 5)	5	0
Subnetworks 14, 22 & 26 (n = 3) (one review/network)	3	0
Total	14 (100%)	0 (0%)
No known Alcohol Industry funding histories		
Subnetwork 1 (n = 12)	4	8
Subnetwork 4 (n = 4)	3	1
Subnetwork 5 (n = 3)	0	2
Subnetwork 6 (n = 2)	1	1
Subnetwork 7 (n = 2)	1	1
Subnetwork 8 (n = 2)	2	0
Subnetwork 9 (n = 2)	1	1
Subnetworks 10–13, 15–21, 23–25, 27–31 (n = 19) (one review/network)	13	5
Total	25 (54%)	19 (41%)

NB: Two reviews with no known histories of alcohol industry funding were inconclusive (one from subnetwork 5, the other an isolate review).

## 4 Co-authorship networks over time

To understand how this literature has accumulated over time, we explored changes in co-authorship networks, calculating metrics at 5 year intervals (Table 4). These show that researchers have become more willing to collaborate, with the density of the overall network reducing as reviews of CVD and alcohol increase, with fewer new co-authorships added in comparison.

**Table 4:** Cumulative structural and network metrics over time.

Year	2000	2005	2010	2015	2020
Total SRs	5	10	20	42	60
Total Authors	15	38	62	149	231
Average SRs/author	1.133	1.105	1.435	1.349	1.329
Average authors/SR	3.4 (17/5)	4.2 (42/10)	4.5 (89/20)	4.8 (201/42)	5.1 (307/60)
Co-authorships	26	78	138	442	713
Network Density	0.248	0.111	0.073	0.040	0.027
Conclusions of protective effect	4 (80%)	10 (60%)	13 (65%)	29 (70%)	39 (65%)
Alcohol industry funding author histories	3 (60%)	4 (40%)	9 (45%)	13 (31%)	14 (23%)
Number of subnetworks	3	7	13	24	31
Subnetworks with alcohol industry funding histories	2	3	5	5	5

NB Network Density refers to the number of observed connections relative to the number of possible connections. The more dense the network the more connected it is.

Authorship of reviews by colleagues with histories of alcohol industry funding have become less prominent over time. This is in part due to the increase in the number of isolate reviews, and in part because some subnetworks have amalgamated, with two subnetworks with author industry funding histories joined by Bagnardi in 2008, and another two by Rimm in 2016.

### 4.1 Comparison of reviews with and without any known alcohol industry funding history

Reviews with and without any known alcohol industry funding history are very different from each other in what they review. Studies undertaken by authors with industry funding histories are more likely to study broader cardiovascular disease or coronary heart disease as outcomes (93%, 13/14 reviews), whereas studies without such funding histories focus on more specific outcomes, such as hypertension (4 reviews), atrial fibrillation (4 reviews) or stroke (4 reviews) (59%, 27/46). The Chi-squared test statistic is 12.4, p-value <0.001.

They also differ in where they were published, with the industry funding history reviews more likely to be published in general medical journals (43%, 6/14 reviews versus 13%, 6/46 reviews). Reviews with no industry funding associations were more likely to be published in alcohol or addiction journals (22%, 10/46 reviews versus 7%, 1/14 reviews). There were similar proportions published in cardiology journals (21%, 3/14 reviews versus 24%, 11/46 reviews) and epidemiology or public health journals (14%, 2/14 reviews versus 15%, 7/46 reviews).

The mean number of authors in the two groups of reviews is similar (5.36, range 3–12 versus 5.04, range 2–13), as was the mean number of systematic reviews published per author (3.5 versus 4.0, with and without industry funding histories). Extent of referencing in the introductions and discussions is similar in both categories of reviews (37 (range 13–66) versus 39 range 15–101) with and without industry funding histories). The mean number of included studies was higher in the industry funding history group; (29, (range 6–84) versus n = 20 (range 6–45), though this difference was not statistically significant (t = 1.23, p = 0.238).

### 4.2 Citations

There was a large significant difference in the mean number of citations between the two funding categories (industry funding history 327 (range 16–927) versus 85 (range 0–556) no industry funding history (Mann-Whitney test, z = −3.157, p = 0.0012). This difference is greater than that when one compares the mean number of citations by direction of the conclusions (168 for cardioprotection conclusion versus 98 for no cardioprotection).

The reviews by authors with alcohol industry funding histories are older. When mean citations per year are investigated, the difference is attenuated, though remains clear 21 (95% CI 13, 28) versus 9 (95% CI 7, 11), t = 2.8, p = 0.012).

**Fig. 2:**
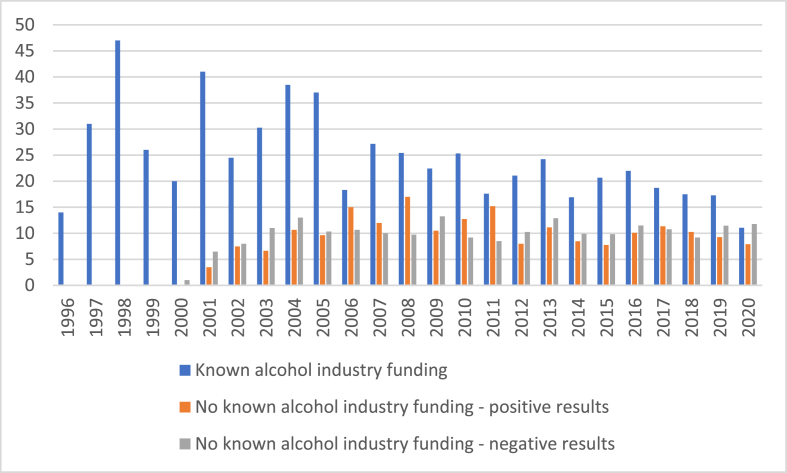
Mean number of times systematic reviews cited in each year.

Fig. 2 shows the mean number of times reviews have been cited in each year, and clearly demonstrates that reviews associated with industry funding, independently of publication year and cardioprotection conclusions, were cited more often. There is no strong pattern of citations for reviews that are free of industry associations, with some evidence suggesting that reviews with cardioprotective conclusions were more likely to be cited up to 2011, with conclusions of no cardioprotection somewhat more likely to be cited since 2012. In addition, reviews by authors with industry funding histories are less frequently cited over time.

## 5 Discussion

Nearly a quarter (23%, 14/60) of systematic reviews undertaken on the impact of alcohol on CVD had a known connection to alcohol industry funding. These formed distinct co-authorship subnetworks within the literature. All reviews by authors with histories of alcohol industry support identified a health protective effect of alcohol, whereas those with no known history of support were approximately evenly divided. The reviews associated with industry were more likely to study broader CVD outcomes, as opposed to more specific CVD outcomes such as hypertension or stroke, had a higher number of included studies and were more influential, being more likely to be cited by others. The reasoning behind the selection of broader outcomes by alcohol industry associated authors is beyond the scope of this study. It can be noted, however, that high quality scientific study of such outcomes may be more attractive for publication in general medical journals. At the same time, industry actors may possess a different set of motivations for such study. Over time the proportion of systematic reviews on CVD and alcohol authored by those with histories of funding by industry has declined, and there has been an increase in reviews more likely to conclude there is no evidence for cardioprotection.

We used declarations of funding and conflicts of interest statements in the published systematic reviews and in other work by the authors as indexed in the suite of databases in the Web of Science (Golder, 2020). Although this novel approach is a major strength of this study, as it averts sole reliance on declarations made for the review, it is likely that we will have missed any funding which was deliberately concealed, as was the case with the tobacco industry (Oreskes and Conway, 2010, Zhang et al., 2015). The findings of this study also draw attention to the possible limitations of declarations of conflicts of interest within relatively short timeframes, such as three years.

Our approach is binary in respect of any prior history of industry funding. This limitation means we have not studied the extent or recency of industry funding or other kinds of relationships with alcohol companies or related organisations. By selecting systematic reviews of CVD only for study we did not investigate links between authors on primary studies of CVD or on reviews of other outcomes (such as diabetes or cancer). Solely relying on the peer-reviewed database sources, as we did, entails that there may be grey literature that is relevant to the aims of this study.

There have been few similar studies, as there has not been any tradition of empirical research on alcohol industry funding effects, or other aspects of involvement in the production of scientific evidence (McCambridge and Mialon, 2018). In light of the damage alcohol does to global health, this study thus makes an important contribution to a very much under-developed literature. It may also assist efforts to resolve the decades-long controversy about alcohol and CVD, and provides data that strongly refutes the industry claim that industry funding is not associated with health outcomes in meta-analytic studies, by adopting a broader perspective on how industry funding may confer bias. It also draws attention to the need for further investigation of the existing controversies themselves. The MACH trial report (Spiegelman et al., 2020) provides scant information on the reasons why the trial was stopped. The authors have also not, to our knowledge, responded to a request (Mitchell et al., 2020) to make a statement on conflict of interest in the paper published that makes the scientific case for the study (Mukamal et al., 2016).

This study raises difficult issues, which have not been widely discussed or well-studied for alcohol, but which are too important to continue to ignore. The alcohol industry has been able to fund researchers in ways that are no longer possible for tobacco companies (Babor and Robaina, 2013). The tobacco and alcohol industries are connected in multiple ways, including through co-ownership (Bond et al., 2009), and continue to collaborate in influencing public policy (McCambridge et al., 2019). This means that it is appropriate to regard the alcohol research literature as having potentially been biased in similar ways to the tobacco literature. (Bero, 2003; Godlee et al., 2013) The study of such bias in the absence of internal documents may be challenging, as it is cumulative over time and hampered by earlier weaknesses in norms relating to declarations of interest. The same is true of funding effects subtly manufactured by pharmaceutical corporations (Smith, 2005), and evidence-based advances have been made in that area.

This study does not provide proof of bias. The differences between the findings of the two sets of reviews requires more in-depth study capable of interrogating how far differences in the detail of design and conduct may account for the observed discrepancies. The authors of these reviews are themselves well positioned to contribute to such study. Bias may operate through the entire research process (Odierna et al., 2013). For instance, the selection of conditions and meaningful outcomes is important in systematic reviews. The selection of wide outcomes such as all-cause mortality for example, has been argued to render reviews meaningless (Rehm, 2019). We suggest further study of individual reviews is not likely to be well served by conventional risk of bias tools, and we have no particular reason to anticipate differences in the findings of such appraisals between the two sets of reviews considered here. More subtle and profound threats posed by industry influence on research agendas, in research careers and among networks are intrinsically challenging to capture (Fabbri et al., 2018; Mitchell and McCambridge, 2021a, 2021b). It may be the case that further in-depth study of these reviews may yield explanations for observed findings other than to do with industry funding.

This study was also not designed to answer questions about whether alcohol may benefit CVD. The present findings suggest that such efforts should be intensified, perhaps with publicly funded trials undertaken entirely independently from any industry influence. Further investigations of differences between the two groups of reviews identified here, and the primary literature they draw on, are needed, as they may provide alternative explanations to bias associated with industry funding. One could also undertake further studies on other major conditions (such as diabetes and cancer with known associations with alcohol), as well as studies on the included studies within these 60 systematic reviews on CVD. Another approach to taking forward the research implications would be to investigate whether the authors are funded by other industries. We intend firstly to investigate patterns in the primary CVD literature. We also hope to expand this area of study into other disease areas.

We also need to develop much more fine grained measures of the details of relations between researchers and industry actors that may give rise to such bias. Interview studies with researchers to unravel the complexities of interactions, and explorations of unexplained heterogeneity between the findings of existing studies may both help to generate understanding of the mechanisms and scale of any bias in the alcohol literature. Such data may be anticipated to have wide generalisability in other fields in which powerful commercial actors possess the capacity to shape science to advance commercial interests to the detriment of health, particularly for important health issues that have been insufficiently studied.

The citations data attest to the enduring influence of the idea that alcohol may be good for the heart within scientific communities, as well as having a hold on public and policy perceptions of alcohol. This idea has been assiduously promoted by the alcohol industry (McCambridge et al., 2018, Savell et al., 2016) for whom it looks clearly important to political strategies (McCambridge et al., 2020). This study demonstrates that there is a need not only to resolve the long running controversy, but also to pay attention to the actions of the alcohol industry in influencing the science (McCambridge and Madden, 2021). It is striking how little we know about a subject that does such large and growing damage to global health.

## Ethics approval and consent to participate

Ethics approval for the conduct of this study was not required because all the data analysed are available in the peer reviewed literature.

## Consent for publication

Not applicable.

## Availability of data and materials

There are no data for sharing as the data are already in the peer reviewed literature. All data generated or analysed during this study are included in this published article and supplementary files.

## Funding

This study forms part of a research programme funded by the 10.13039/100010269Wellcome Trust to investigate the public health implications of alcohol industry influence of science and policy. The funder had no role in any aspect of the study or the decision to submit for publication.

## Author contributions

SG and JM jointly had the idea for this study and led all aspects of study design and conduct. Data collection, analysis and writing of the paper was conducted by SG. JM contributed to interpretation and revisions of the paper and data checking. SG is the guarantor.

## Declaration of competing interest

All authors have completed the ICMJE uniform disclosure form at www.icmje.org/coi_disclosure.pdf and declare: JM and SG had financial support from the 10.13039/100010269Wellcome Trust for the submitted work, via an Investigator Award to the former (200321/Z/15/Z); no financial relationships with any organisations that might have an interest in the submitted work in the previous three years; no other relationships or activities that could appear to have influenced the submitted work.
