# Supplementary material for: Alcohol, cardiovascular disease and industry funding: A co-authorship network analysis of systematic reviews
Source: Soc Sci Med. 2021 Nov;289:114450. doi: 10.1016/j.socscimed.2021.114450 (PMC8586735; doi:10.1016/j.socscimed.2021.114450)
Supplement: Multimedia component 1 [file mmc1.docx]

**Supplementary table 1: Alcohol industry funding declarations for authors of the 60 systematic reviews**

Data from updated (November 2020) search of Web of Science databases as reported in Golder S, Garry J, McCambridge J. [Declared funding and authorship by alcohol industry actors in the scientific literature: a bibliometric study.](https://pubmed.ncbi.nlm.nih.gov/32939544/) Eur J Public Health. 2020.

| **Author** | **Type of alcohol industry funding declaration** | **Reference and funding declaration details** |
| --- | --- | --- |
| **SUBNETWORK 1 (Contained 12 systematic reviews)** | | |
| Gerhard Gmel | **Alcohol industry research funding declaration** | None identified |
|  | **Other declarations of alcohol industry support** | None identified |
|  | **Declarations by co-authors** | Plant M, Miller P, Plant M, **Gmel G**, Kuntsche S, Bergmark K, Bloomfield, K. Csemy, L. Ozenturk, T. Vidal, A. The Social Consequences of Binge Drinking Among 24-to 32-Year-Olds in Six European Countries. Substance Use & Misuse. 2010;45(4):528-42.  *This study was devised by the members of the International Research Group on Alcohol and Gender (IRGGA). This group now operates under the acronym "GENACIS." The authors are especially grateful to Professors Sharon and Richard Wilsnack of the University of North Dakota for advice and encouragement.* ***The analysis presented in this paper was supported by a grant to the Alcohol and Health Research Trust (UK) from the European Forum for Responsible Drinking (formerly known as The Amsterdam Group).*** *Additional support was provided by the University of the West of England, Bristol, UK.* ***The funding sources of the individual national studies associated with this paper were as follows:****; The Czech Republic: The Ministry of Health of the Czech Republic (grant MZ 23752).; Denmark: The Sygkassernes Helsefond (journal no. 2003B195) with additional support from the Danish Medical Research Council (contract no. 22-02-374).; Isle of Man: Isle of Man Medical Research and the University of the West of England, Bristol, UK.; Spain: Direccion General de Atencion a la Dependencia, Conselleria de Sanidad, Generalitat Valenciana, Comisionado do Plan de Galicia sobre Drogas, Conselleria de Sanidade, Xunta de Galicia, and Direccion General de Drogodependencias y Servicios Sociales, Gobierno de Cantabria.; Sweden: The Swedish Ministry of Health and Social Affairs.; United Kingdom:* ***The European Forum for Responsible Drinking,*** *the Alcohol Education and Research Council, and the University of the West of England, Bristol, UK.* |
| Jurgen Rehm | **Alcohol industry research funding declaration** | None identified |
|  | **Other declarations of alcohol industry support** | Roerecke M, **Rehm J**. The cardioprotective association of average alcohol consumption and ischaemic heart disease: a systematic review and meta-analysis. Addiction. 2012;107(7):1246-60.  ***J. Rehm has participated in scientific meetings organized or sponsored by the alcohol industry and received financial support for this participation.***  Taylor B, Irving HM, Baliunas D, Roerecke M, Patra J, Mohapatra S, **Rehm J**. Alcohol and hypertension: gender differences in dose-response relationships determined through systematic review and meta-analysis. Addiction. 2009;104(12):1981-90.  ***J. Rehm has participated in scientific meetings organized or sponsored by the alcohol industry and received financial support for this participation.*** |
|  | **Declarations by co-authors** | Vos T, Barber RM, Bell B, Bertozzi-Villa A, Biryukov S, Bolliger I, Charlson, F. Davis, A. Degenhardt, L. Dicker, D. Duan, L. Erskine, H. Feigin, V. L. Ferrari, A. J. Fitzmaurice, C. Fleming, T. Graetz, N. Guinovart, C. Haagsma, J. et al. Global, regional, and national incidence, prevalence, and years lived with disability for 301 acute and chronic diseases and injuries in 188 countries, 1990-2013: a systematic analysis for the Global Burden of Disease Study 2013. Lancet. 2015;386(9995):743-800.  ***DS*** ***has received research grants or consultancy honoraria from Abbott,*** ***ABMRF****, Astrazeneca, Biocodex, Eli-Lilly, GlaxoSmithKline, Jazz Pharmaceuticals, Johnson & Johnson, Lundbeck, National Responsible Gambling Foundation, Novartis, Orion, Pfizer, Pharmacia, Roche, Servier, Solvay, Sumitomo, Sun, Takeda, Tikvah, and Wyeth. DS would like to acknowledge support by the Medical Research Council of South Africa.* |
| Antoni Gual | **Alcohol industry research funding declaration** | None identified |
|  | **Other declarations of alcohol industry support** | None identified |
|  | **Declarations by co-authors** | Calleja-Conde J, Echeverry-Alzate V, Gine E, Buhler KM, Nadal R, Maldonado R, de Fonseca, F. R. **Gual, A.** Lopez-Moreno, J. A.. Nalmefene is effective at reducing alcohol seeking, treating alcohol-cocaine interactions and reducing alcohol-induced histone deacetylases gene expression in blood. British Journal of Pharmacology. 2016;173(16):2490-505.  ***This work was supported by The*** ***European Foundation for Alcohol Research (to J.A.L.M., F.R.d.F., R.M. and R.N.),*** *the Fondo de Investigacion Sanitaria (Red de Trastornos Adictivos, FEDER, RD12/0028/0015 to J.A.L.M., RD12/0028/001 to F.R.d.F., RD12/0028/023 to R.M., RD12/0028/0014 to R.N.) and Ministerio de Ciencia e Innovacion (SAF2011-26818 to J.A.L.M.).* |
| **SUBNETWORK 2 (Contained 6 systematic reviews)** | | |
| Simona Costanzo | **Alcohol industry research funding declaration** | **Costanzo S**, Di Castelnuovo A, Donati MB, Iacoviello L, de Gaetano G. Wine, beer or spirit drinking in relation to fatal and non-fatal cardiovascular events: a meta-analysis. Eur J Epidemiol. 2011;26(11):833-50.  ***Supported in part by Cervisia Consulenze*** *and Istituto Nazionale per la Comunicazione. This was an investigator-initiated study. The partial sponsor of the study had no role in the selection of articles or conduct of the analyses or drafting of the manuscript. We disclaim any other relationships with industry that might pose a conflict of interest in connection with the submitted article.*  **Costanzo S**, Di Castelnuovo A, Donati MB, Iacoviello L, de Gaetano G. Alcohol Consumption and Mortality in Patients With Cardiovascular Disease A Meta-Analysis. Journal of the American College of Cardiology. 2010;55(13):1339-47.  *From the Laboratory of Genetic and Environmental Epidemiology, "RE ARTU" Research Laboratories, "John Paul II" Centre for High Technology Research and Education in Biomedical Sciences, Catholic University, Campobasso, Italy.* ***Supported in part by grant EA0827 from the European Research Advisory Board (ERAB).*** *The authors thank Professor Jozef Vermylen, Catholic University, Leuven, Belgium, for his critical review of the manuscript; Dr. Vincenzo Bagnardi, University of Milan Bicocca, Milan, Italy, for providing the macro SAS; and Ms. Judith Baggott for English editing*.  **Costanzo S,** De Curtis A, di Niro V, Olivieri M, Morena M, De Filippo CM, Caradonna, E. Krogh, V. Serafini, M. Pellegrini, N. Donati, M. B. de Gaetano, G. Iacoviello, L. Postoperative atrial fibrillation and total dietary antioxidant capacity in patients undergoing cardiac surgery: The Polyphemus Observational Study. Journal of Thoracic and Cardiovascular Surgery. 2015;149(4):1175-82.  ***This study was supported by*** *the Italian Ministry of Health, Young Researchers Grant Number 2008-1146478,* ***The European Foundation for Alcohol Research (ERAB) Grant EA082****, The* ***International Organisation of Vine and Wine (OIV)*** ***Grant 2011 to*** ***S.C****., and the Project "Malattie cardiovascolari: ruoli di fattori genetici, acquisiti, nuovi approcci terapeutici e condizioni organizzative ottimali per la produzione delle conoscenze'' (D. MIUR n. 328 del 01/07/2010). These funding sources had no involvement in the study design; the collection, analysis, and interpretation of data; the writing of the article; or the authors' decision to submit the article for publication.*  **Costanzo S,** Di Castelnuovo A, Donati MB, Iacoviello L, de Gaetano G. Alcohol Consumption and Mortality in Patients With Cardiovascular Disease A Meta-Analysis. Journal of the American College of Cardiology. 2010;55(13):1339-47.  *From the Laboratory of Genetic and Environmental Epidemiology, "RE ARTU" Research Laboratories, "John Paul II" Centre for High Technology Research and Education in Biomedical Sciences, Catholic University, Campobasso, Italy.* ***Supported in part by grant EA0827 from the*** ***European Research Advisory Board (ERAB).*** *The authors thank Professor Jozef Vermylen, Catholic University, Leuven, Belgium, for his critical review of the manuscript; Dr. Vincenzo Bagnardi, University of Milan Bicocca, Milan, Italy, for providing the macro SAS; and Ms. Judith Baggott for English editing.*  **Costanzo S,** Di Castelnuovo A, Donati MB, Iacoviello L, de Gaetano G. Cardiovascular and Overall Mortality Risk in Relation to Alcohol Consumption in Patients With Cardiovascular Disease. Circulation. 2010;121(17):1951-9.  ***This study was funded in part by a grant from the*** ***European Research Advisory Board (No. EA0827).***  de Gaetano G, **Costanzo S**, Di Castelnuovo A, Badimon L, Bejko D, Alkerwi A, Chiva-Blanch, G. Estruch, R. La Vecchia, C. Panico, S. Pounis, G. Sofi, F. Stranges, S. Trevisan, M. Ursini, F. Cerletti, C. Donati, M. B. Iacoviello, L. Effects of moderate beer consumption on health and disease: A consensus document. Nutrition Metabolism and Cardiovascular Diseases. 2016;26(6):443-67. ***Giovanni de Gaetano*** *is a consultant to the Web Newsletter of* ***Assobirra,*** *the Italian Association of the Beer and Malt Industries; Simona Costanzo: none; Augusto Di Castelnuovo: none; Ala’a Alkerwi: none;* ***Lina Badimon*** *is a Member of the Advisory Board of* ***Fundación Cerveza y Salud****; Dritan Bejko: none; Gemma Chiva-Blanch: none;* ***Ramon Estruch*** *is serving on the board of and receiving lecture fees from the* ***Research Foundation on Wine and Nutrition (FIVIN);*** *serving on the boards of the* ***Beer and Health Foundation*** *and the* ***European Foundation for Alcohol Research (ERAB);*** *receiving lecture fees from* ***Cerveceros de España****; Carlo La Vecchia: none; Salvatore Panico: none; George Pounis: none; Francesco Sofi: none; Saverio Stranges: none; Maurizio Trevisan: none;* ***Fulvio Ursini*** *is a consultant to the Web Newsletter of* ***Assobirra,*** *the Italian Association of the Beer and Malt Industries; Chiara Cerletti: none; Maria Benedetta Donati: none; Licia Iacoviello: none.* ***This study was supported in part by Assobirra, the Italian Association of the Beer and Malt Industries.*** *This funding source had no involvement in either study design, or selection of the Panel members, or collection and interpretation of data, or the writing of the report nor in Panel’s decision to submit the manuscript for publication. We disclaim any other relationships with any industry or individuals who that might pose a conflict of interest in connection with the submitted article. The manuscript has been read and approved for submission to Nutrition, Metabolism and Cardiovascular Disease by all Authors.*  Koch M, **Costanzo S,** Fitzpatrick AL, Lopez OL, DeKosky S, Kuller LH, Price, J. Mackey, R. H. Jensen, M. K. Mukamal, K. J. Alcohol Consumption, Brain Amyloid-beta Deposition, and Brain Structural Integrity Among Older Adults Free of Dementia. J Alzheimers Dis. 2020;74(2):509-19.  *Samples from the National Cell Repository for Alzheimer's Disease (NCRAD), which receives government support under a cooperative agreement grant (U24 AG21886) awarded by the National Institute on Aging (NIA), were used in this study. This work was supported by the NIH/NINDS (1R01NS089638-01A1).* ***Simona Costanzo is principal investigator of an ongoing study supported by a research grant from the*** ***European Foundation for Alcohol Research (ERAB,*** *EA1767). The funding sources had no role in study design; in the collection, analysis and interpretation of data; in the writing of the report; and in the decision to submit the article for publication.* |
|  | **Other declarations of alcohol industry support** | ***Simona Costanzo*** *is on the 9th* ***Beer and Health*** *Symposium Organising Committee https://beerandhealth.eu/symposium/organising-committee/costanzo-simona/ The Beer and Health initiative is supported financially by The Brewers of Europe. The realization of Birrainforma, whose management is autonomous, is made possible thanks to the external support of Assobirra https://beerandhealth.eu/scientific-committee/* |
|  | **Declarations by co-authors** | None identified |
| Augusto Di Castelnuovo | **Alcohol industry research funding declaration** | Costanzo S, **Di Castelnuovo A,** Donati MB, Iacoviello L, de Gaetano G. Wine, beer or spirit drinking in relation to fatal and non-fatal cardiovascular events: a meta-analysis. Eur J Epidemiol. 2011;26(11):833-50.  ***Supported in part by*** ***Cervisia Consulenze*** *and Istituto Nazionale per la Comunicazione. This was an investigator-initiated study. The partial sponsor of the study had no role in the selection of articles or conduct of the analyses or drafting of the manuscript. We disclaim any other relationships with industry that might pose a conflict of interest in connection with the submitted article.*  Costanzo S, **Di Castelnuovo A,** Donati MB, Iacoviello L, de Gaetano G. Alcohol Consumption and Mortality in Patients With Costanzo S, Di Castelnuovo A, Donati MB, Iacoviello L, de Gaetano G. Alcohol Consumption and Mortality in Patients With Cardiovascular Disease A Meta-Analysis. Journal of the American College of Cardiology. 2010;55(13):1339-47.  *From the Laboratory of Genetic and Environmental Epidemiology, "RE ARTU" Research Laboratories, "John Paul II" Centre for High Technology Research and Education in Biomedical Sciences, Catholic University, Campobasso, Italy.* ***Supported in part by grant EA0827 from the*** ***European Research Advisory Board (ERAB).*** *The authors thank Professor Jozef Vermylen, Catholic University, Leuven, Belgium, for his critical review of the manuscript; Dr. Vincenzo Bagnardi, University of Milan Bicocca, Milan, Italy, for providing the macro SAS; and Ms. Judith Baggott for English editing*.  Costanzo S, **Di Castelnuovo A,** Donati MB, Iacoviello L, de Gaetano G. Alcohol Consumption and Mortality in Patients With Cardiovascular Disease A Meta-Analysis. Journal of the American College of Cardiology. 2010;55(13):1339-47.  *From the Laboratory of Genetic and Environmental Epidemiology, "RE ARTU" Research Laboratories, "John Paul II" Centre for High Technology Research and Education in Biomedical Sciences, Catholic University, Campobasso, Italy. Supported in part by grant EA0827 from the* ***European Research Advisory Board (ERAB).*** *The authors thank Professor Jozef Vermylen, Catholic University, Leuven, Belgium, for his critical review of the manuscript; Dr. Vincenzo Bagnardi, University of Milan Bicocca, Milan, Italy, for providing the macro SAS; and Ms. Judith Baggott for English editing.*  Costanzo S, **Di Castelnuovo A,** Donati MB, Iacoviello L, de Gaetano G. Cardiovascular and Overall Mortality Risk in Relation to Alcohol Consumption in Patients With Cardiovascular Disease. Circulation. 2010;121(17):1951-9.  ***This study was funded in part by a grant from the European Research Advisory Board (No. EA0827).***  di Giuseppe R, de Lorgeril M, Salen P, Laporte F, **Di Castelnuovo** A, Krogh V, Siani, A. Arnout, J. Cappuccio, F. P. van Dongen, M. Donati, M. B. de Gaetano, G. Iacoviello, L. Alcohol consumption and n-3 polyunsaturated fatty acids in healthy men and women from 3 European populations. American Journal of Clinical Nutrition. 2009;89(1):354-62.  ***Supported by the*** ***European Research Advisory Board (grant EA 05 20)*** *and the European Union (grant QLK1-2000-00100). MIUR (Ministero dell'Istruzione, Universita e Ricerca, Italia) Programma Triennale di Ricerca (grant D. 1588), and the Fondazione Invernizzi are currently supporting GdG and his associates at the Catholic University in Campobasso, Italy.*  Latella MC, **Di Castelnuovo A,** de Lorgeril M, Arnout J, Cappuccio FP, Krogh V, Siani, A. van Dongen, M. Donati, M. B. de Gaetano, G. Iacoviello, L. Genetic variation of alcohol dehydrogenase type 1C (ADH1C), alcohol consumption, and metabolic cardiovascular risk factors: Results from the IMMIDIET study. Atherosclerosis. 2009;207(1):284-90.  ***The IMMIDIET study was supported by*** *the European Union grant no. QLK1-2000-00100. MIUR (Ministero dell'Universita e Ricerca, Italia)-Programma Triennale di Ricerca (grant D. 1588)* ***ERAB ( grant EA 05 20)*** *and Fondazione Invernizzi supported L. I. and her associates at the Catholic University in Campobasso, Italy.*  de Gaetano G, Costanzo S, **Di Castelnuovo A,** Badimon L, Bejko D, Alkerwi A, Chiva-Blanch, G. Estruch, R. La Vecchia, C. Panico, S. Pounis, G. Sofi, F. Stranges, S. Trevisan, M. Ursini, F. Cerletti, C. Donati, M. B. Iacoviello, L. of moderate beer consumption on health and disease: A consensus document. Nutrition Metabolism and Cardiovascular Diseases. 2016;26(6):443-67.  ***Giovanni de Gaetano*** *is a consultant to the Web Newsletter of* ***Assobirra****, the Italian Association of the Beer and Malt Industries; Simona Costanzo: none; Augusto Di Castelnuovo: none; Ala’a Alkerwi: none;* ***Lina Badimon*** *is a Member of the Advisory Board of* ***Fundación Cerveza y Salud****; Dritan Bejko: none; Gemma Chiva-Blanch: none;* ***Ramon Estruch*** *is serving on the board of and receiving lecture fees from the* ***Research Foundation on Wine and Nutrition (FIVIN);*** *serving on the boards of the* ***Beer and Health Foundation*** *and the* ***European Foundation for Alcohol Research (ERAB);*** *receiving lecture fees from* ***Cerveceros de España****; Carlo La Vecchia: none; Salvatore Panico: none; George Pounis: none; Francesco Sofi: none; Saverio Stranges: none; Maurizio Trevisan: none;* ***Fulvio Ursini*** *is a consultant to the Web Newsletter of* ***Assobirra****, the Italian Association of the Beer and Malt Industries; Chiara Cerletti: none; Maria Benedetta Donati: none; Licia Iacoviello: none.* ***This study was supported in part by Assobirra, the Italian Association of the Beer and Malt Industries.*** *This funding source had no involvement in either study design, or selection of the Panel members, or collection and interpretation of data, or the writing of the report nor in Panel’s decision to submit the manuscript for publication. We disclaim any other relationships with any industry or individuals who that might pose a conflict of interest in connection with the submitted article. The manuscript has been read and approved for submission to Nutrition, Metabolism and Cardiovascular Disease by all Authors.* |
|  | **Other declarations of alcohol industry support** | None identified |
|  | **Declarations by co-authors** | None identified |
| Giovanni de Gaetano | **Alcohol industry research funding declaration** | Costanzo S, Di Castelnuovo A, Donati MB, Iacoviello L**, de Gaetano G.** Wine, beer or spirit drinking in relation to fatal and non-fatal cardiovascular events: a meta-analysis. Eur J Epidemiol. 2011;26(11):833-50.  ***Supported in part by*** ***Cervisia Consulenze*** *and Istituto Nazionale per la Comunicazione. This was an investigator-initiated study. The partial sponsor of the study had no role in the selection of articles or conduct of the analyses or drafting of the manuscript. We disclaim any other relationships with industry that might pose a conflict of interest in connection with the submitted article.*  Costanzo S, Di Castelnuovo A, Donati MB, Iacoviello L, **de Gaetano G.** Alcohol Consumption and Mortality in Patients With Cardiovascular Disease A Meta-Analysis. Journal of the American College of Cardiology. 2010;55(13):1339-47.  *From the Laboratory of Genetic and Environmental Epidemiology, "RE ARTU" Research Laboratories, "John Paul II" Centre for High Technology Research and Education in Biomedical Sciences, Catholic University, Campobasso, Italy.* ***Supported in part by grant EA0827 from the*** ***European Research Advisory Board (ERAB).*** *The authors thank Professor Jozef Vermylen, Catholic University, Leuven, Belgium, for his critical review of the manuscript; Dr. Vincenzo Bagnardi, University of Milan Bicocca, Milan, Italy, for providing the macro SAS; and Ms. Judith Baggott for English editing.*  Costanzo S, De Curtis A, di Niro V, Olivieri M, Morena M, De Filippo CM, Caradonna, E. Krogh, V. Serafini, M. Pellegrini, N. Donati, M. B. **de Gaetano,** G. Iacoviello, L. Postoperative atrial fibrillation and total dietary antioxidant capacity in patients undergoing cardiac surgery: The Polyphemus Observational Study. Journal of Thoracic and Cardiovascular Surgery. 2015;149(4):1175-82.  ***This study was supported by*** *the Italian Ministry of Health, Young Researchers Grant Number 2008-1146478,* ***The*** ***European Foundation for Alcohol Research (ERAB) Grant EA082****, The* ***International Organisation of Vine and Wine (OIV)*** *Grant 2011 to* ***S.C****., and the Project "Malattie cardiovascolari: ruoli di fattori genetici, acquisiti, nuovi approcci terapeutici e condizioni organizzative ottimali per la produzione delle conoscenze'' (D. MIUR n. 328 del 01/07/2010). These funding sources had no involvement in the study design; the collection, analysis, and interpretation of data; the writing of the article; or the authors' decision to submit the article for publication.*  Costanzo S, Di Castelnuovo A, Donati MB, Iacoviello L, **de Gaetano G.** Alcohol Consumption and Mortality in Patients With Cardiovascular Disease A Meta-Analysis. Journal of the American College of Cardiology. 2010;55(13):1339-47.  *From the Laboratory of Genetic and Environmental Epidemiology, "RE ARTU" Research Laboratories, "John Paul II" Centre for High Technology Research and Education in Biomedical Sciences, Catholic University, Campobasso, Italy.* ***Supported in part by grant EA0827 from the*** ***European Research Advisory Board (ERAB).*** *The authors thank Professor Jozef Vermylen, Catholic University, Leuven, Belgium, for his critical review of the manuscript; Dr. Vincenzo Bagnardi, University of Milan Bicocca, Milan, Italy, for providing the macro SAS; and Ms. Judith Baggott for English editing.*  Costanzo S, Di Castelnuovo A, Donati MB, Iacoviello L, **de Gaetano G.** Cardiovascular and Overall Mortality Risk in Relation to Alcohol Consumption in Patients With Cardiovascular Disease. Circulation. 2010;121(17):1951-9.  ***This study was funded in part by a grant from the*** ***European Research Advisory Board (No. EA0827).***  di Giuseppe R, de Lorgeril M, Salen P, Laporte F, Di Castelnuovo A, Krogh V, Siani, A. Arnout, J. Cappuccio, F. P. van Dongen, M. Donati, M. B. **de Gaetano,** G. Iacoviello, L. Alcohol consumption and n-3 polyunsaturated fatty acids in healthy men and women from 3 European populations. American Journal of Clinical Nutrition. 2009;89(1):354-62**.**  ***Supported by the*** ***European Research Advisory Board (grant EA 05 20)*** *and the European Union (grant QLK1-2000-00100). MIUR (Ministero dell'Istruzione, Universita e Ricerca, Italia) Programma Triennale di Ricerca (grant D. 1588), and the Fondazione Invernizzi are currently supporting GdG and his associates at the Catholic University in Campobasso, Italy*.  Latella MC, Di Castelnuovo A, de Lorgeril M, Arnout J, Cappuccio FP, Krogh V, Siani, A. van Dongen, M. Donati, M. B. **de Gaetano, G**. Iacoviello, L. Genetic variation of alcohol dehydrogenase type 1C (ADH1C), alcohol consumption, and metabolic cardiovascular risk factors: Results from the IMMIDIET study. Atherosclerosis. 2009;207(1):284-90.  ***The IMMIDIET study was supported by*** *the European Union grant no. QLK1-2000-00100. MIUR (Ministero dell'Universita e Ricerca, Italia)-Programma Triennale di Ricerca ( grant D. 1588)* ***ERAB ( grant EA 05 20)*** *and Fondazione Invernizzi supported L. I. and her associates at the Catholic University in Campobasso, Italy.*  **de Gaetano G,** Costanzo S, Di Castelnuovo A, Badimon L, Bejko D, Alkerwi A, Chiva-Blanch, G. Estruch, R. La Vecchia, C. Panico, S. Pounis, G. Sofi, F. Stranges, S. Trevisan, M. Ursini, F. Cerletti, C. Donati, M. B. Iacoviello, L.. Effects of moderate beer consumption on health and disease: A consensus document. Nutrition Metabolism and Cardiovascular Diseases. 2016;26(6):443-67. ***Giovanni de Gaetano is a consultant to the Web Newsletter of Assobirra, the Italian Association of the Beer and Malt Industries****; Simona Costanzo: none; Augusto Di Castelnuovo: none; Ala’a Alkerwi: none;* ***Lina Badimon*** *is a Member of the Advisory Board of* ***Fundación Cerveza y Salud****; Dritan Bejko: none; Gemma Chiva-Blanch: none;* ***Ramon Estruch*** *is serving on the board of and receiving lecture fees from the* ***Research Foundation on Wine and Nutrition (FIVIN****); serving on the boards of the* ***Beer and Health Foundation and the European Foundation for Alcohol Research (ERAB);*** *receiving lecture fees from Cerveceros de España; Carlo La Vecchia: none; Salvatore Panico: none; George Pounis: none; Francesco Sofi: none; Saverio Stranges: none; Maurizio Trevisan: none;* ***Fulvio Ursini*** *is a consultant to the Web Newsletter of* ***Assobirra****, the Italian Association of the Beer and Malt Industries; Chiara Cerletti: none; Maria Benedetta Donati: none; Licia Iacoviello: none.* ***This study was supported in part by Assobirra, the Italian Association of the Beer and Malt Industries.*** *This funding source had no involvement in either study design, or selection of the Panel members, or collection and interpretation of data, or the writing of the report nor in Panel’s decision to submit the manuscript for publication. We disclaim any other relationships with any industry or individuals who that might pose a conflict of interest in connection with the submitted article. The manuscript has been read and approved for submission to Nutrition, Metabolism and Cardiovascular Disease by all Authors.*  Barrett-Connor E, **de Gaetano G,** Djousse L, Ellison RC, Estruch R, Finkel H, Goldfinger, T. Keil, U. Lanzmann-Petithory, D. Mattivi, F. Skovenborg, E. Stockley, C. Svilaas, A. Teissedre, P. L. Thelle, D. S. Ursini, F. Waterhouse, A. L. Comments on Moderate Alcohol Consumption and Mortality. Journal of Studies on Alcohol and Drugs. 2016;77(5):834-6.  ***Giovanni de Gaetano received reimbursement for travel and other organizational costs incurred in the preparation of a consensus document on beer and health that were paid by a research grant from Epicomed Research srl, a small Italian company that had received financial support from*** ***Assobirra, an Italian association of beer industrie****s.;* ***Luc Djousse*** *has received grant funding from the* ***Alcoholic Beverage Medical Research Foundation****.; R.* ***Curtis Ellison*** *was until 2013 (when the Institute ceased operations) the director of the Institute on Lifestyle & Health at Boston University School of Medicine, which received partial support from the* ***wine or beverage alcohol industry****. These were unrestricted donations to Boston University to support the work of the Institute in monitoring the scientific literature on alcohol and health and providing commentaries. Donors had no input into the commentaries. Unrestricted donations received between 2007 and 2013 from the* ***New York Wine & Grape Foundation, Canandaigua, NY; Diageo, Inc., New York City; and Brown-Forman Corporation, Louisville, KY.*** *In 2010,* ***Dr. Ellison*** *became a founder and Scientific Co-Director of the* ***International Scientific Forum on Alcohol Research****. Some* ***beverage industry funds*** *were used in establishing the website of this Forum (*[*www.bu.edu/alcohol-forum*](http://www.bu.edu/alcohol-forum)*). Over the past 50 years, he has been invited to give presentations at more than 100 local, regional, national, or international scientific meetings, many related to alcohol consumption, for which companies in the* ***wine industry*** *provided at least some of the support for travel expenses, including the* ***International Organisation of Vine and Wine, the Distilled Spirits Council of the United States (DISCUS), and the Washington State Grape Society****. In 2006, he helped organize an international scientific conference sponsored in part by the* ***International Center for Alcohol Policies (ICAP),*** *which at the time was funded by a consortium of major* ***alcohol producers and related organizations****.;* ***Dominique Lanzmann****-Petithory has accepted travel funds from the Renaud Society (an International Society of Medical Professionals with an Interest in Better Health and a Passion for Wine) to attend two scientific Meetings, supported in part by* ***wine producers.*** *She was the coordinator of the French National Research Program CANCERALCOOL, financed in part by the* ***Bordeaux Wine Inter-Professional Committee****.;* ***Erik Skovenborg*** *received reimbursement for attending the international "Health & Alcohol Symposium" in Copenhagen on November 8, 1996; the international "Women and Alcohol Symposium" in Stockholm on October 30, 1998; and the international symposium "Alcohol in moderation Health and Culture in the next millennium" in Oslo on October 27, 2000. The reimbursement was paid by GODA, a Danish organization that is funded by* ***Wine and Spirits Companies*** *and also (at that time) the* ***Brewers****. He was also a Member of the Board of Directors of* ***ERAB The European Foundation for Alcohol Research*** *from 2004 to 2016 (no fees or honoraria of any kind) and received reimbursement for attending three conferences on alcohol and health.* ***ERAB*** *and the alcohol and health conferences are sponsored by the* ***Brewers of Europe****. Reimbursement was also received for attending and speaking (no speaker's fee) at the 1st European Conference for Wine Education at the Weinakademie Osterreich/Austrian Wine Academy in Rust/Austria 2008. The* ***Austrian Wine Marketing Board*** *supported the conference.* ***Dr. Skovenborg*** *has occasionally written articles for* ***wine magazines and beer magazines*** *with comments on alcohol and health issues, for which he has received modest payments.;* ***Creina S. Stockley*** *is employed by* ***The Australian Wine Research Institute (AWRI)****, an independent, accredited scientific research institution whose board comprises industry-elected directors, the majority of whom are grape growers, viticulturists, winemakers, or wine industry consultants. The AWRI derives approximately two thirds of its funds from a statutory body, Wine Australia, which is funded from levies on tonnage for grapes that are processed into wine. Additional sources of funds have come from the Foundation for Alcohol Research and Education.; Pierre-Louis Teissedre was, until 2005, a professor at the Institut des Sciences de la Vigne et du Vin, University of Bordeaux, which received support from groups, associations, and companies in the* ***wine industry*** *to support the work of the Institute in monitoring the scientific literature on wine quality as well as health. Donors had no input into the commentaries. He has been invited to give presentations at scientific meetings related to wine and health where travel expenses were paid by the* ***International Organisation of Vine and Wine. Andrew L. Waterhouse****is a faculty member of the Department of Viticulture and Enology at the University of California since 1991. His research program has been largely supported by the* ***wine industry****, either directly from winemaking companies, associations thereof, or affiliated companies that produce products or services used in wine production.* |
|  | **Other declarations of alcohol industry support** | None identified |
|  | **Declarations by co-authors** | Sluik D, Jankovic N, Hughes M, O'Doherty MG, Schottker B, Drygas W, Rolandsson, O. Maennistoe, S. Ordonez-Mena, J. M. Ferrieres, J. Bamia, C. **De Gaetano, G.** Kiefte-De Jong, J. C. Franco, O. H. Sluijs, I. Spijkerman, A. M. W. Sans, S. Eriksson, S. Kromhout, D. Trichopoulou, A. Wilsgaard, T. Brenner, H. Kuulasmaa, K. Laatikainen, T. Soederberg, S. Iacoviello, L. Boffetta, P. Kee, F. Feskens, E. J. M. Alcoholic beverage preference and diabetes incidence across Europe: the Consortium on Health and Ageing Network of Cohorts in Europe and the United States (CHANCES) project. European Journal of Clinical Nutrition. 2017;71(5):659-68.  *This analysis was part of the Consortium on Health and Ageing (CHANCES) project funded in the FP7 framework programme of the Directorate-General for Research and Innovation in the European Commission (grant 242244). The CHANCES project is coordinated by the Hellenic Health Foundation, Greece. Harmonization of the data from the MORGAM cohorts was also supported by European Union FP 7 project BiomarCaRE (278913)****. The research of D Sluik was supported by the Dutch Beer Institute and the European Foundation for Alcohol Research (ERAB).*** |
| Licia Iacoviello | **Alcohol industry research funding declaration** | Costanzo S, Di Castelnuovo A, Donati MB**, Iacoviello L,** de Gaetano G. Wine, beer or spirit drinking in relation to fatal and non-fatal cardiovascular events: a meta-analysis. Eur J Epidemiol. 2011;26(11):833-50.  ***Supported in part by*** ***Cervisia Consulenze*** *and Istituto Nazionale per la Comunicazione. This was an investigator-initiated study. The partial sponsor of the study had no role in the selection of articles or conduct of the analyses or drafting of the manuscript. We disclaim any other relationships with industry that might pose a conflict of interest in connection with the submitted article.*  Costanzo S, Di Castelnuovo A, Donati MB, **Iacoviello L,** de Gaetano G. Alcohol Consumption and Mortality in Patients With Cardiovascular Disease A Meta-Analysis. Journal of the American College of Cardiology. 2010;55(13):1339-47.  *From the Laboratory of Genetic and Environmental Epidemiology, "RE ARTU" Research Laboratories, "John Paul II" Centre for High Technology Research and Education in Biomedical Sciences, Catholic University, Campobasso, Italy.* ***Supported in part by grant EA0827 from the European Research Advisory Board (ERAB).*** *The authors thank Professor Jozef Vermylen, Catholic University, Leuven, Belgium, for his critical review of the manuscript; Dr. Vincenzo Bagnardi, University of Milan Bicocca, Milan, Italy, for providing the macro SAS; and Ms. Judith Baggott for English editing.*  Costanzo S, De Curtis A, di Niro V, Olivieri M, Morena M, De Filippo CM, Caradonna, E. Krogh, V. Serafini, M. Pellegrini, N. Donati, M. B. de Gaetano, G**. Iacoviello, L.** Postoperative atrial fibrillation and total dietary antioxidant capacity in patients undergoing cardiac surgery: The Polyphemus Observational Study. Journal of Thoracic and Cardiovascular Surgery. 2015;149(4):1175-82.  ***This study was supported by*** *the Italian Ministry of Health, Young Researchers Grant Number 2008-1146478,* ***The European Foundation for Alcohol Research (ERAB)*** *Grant EA082, The* ***International Organisation of Vine and Wine (OIV) Grant 2011 to S.C****., and the Project "Malattie cardiovascolari: ruoli di fattori genetici, acquisiti, nuovi approcci terapeutici e condizioni organizzative ottimali per la produzione delle conoscenze'' (D. MIUR n. 328 del 01/07/2010). These funding sources had no involvement in the study design; the collection, analysis, and interpretation of data; the writing of the article; or the authors' decision to submit the article for publication.*  Costanzo S, Di Castelnuovo A, Donati MB, **Iacoviello L,** de Gaetano G. Alcohol Consumption and Mortality in Patients With Cardiovascular Disease A Meta-Analysis. Journal of the American College of Cardiology. 2010;55(13):1339-47.  *From the Laboratory of Genetic and Environmental Epidemiology, "RE ARTU" Research Laboratories, "John Paul II" Centre for High Technology Research and Education in Biomedical Sciences, Catholic University, Campobasso, Italy.* ***Supported in part by grant EA0827 from the*** ***European Research Advisory Board (ERAB).*** *The authors thank Professor Jozef Vermylen, Catholic University, Leuven, Belgium, for his critical review of the manuscript; Dr. Vincenzo Bagnardi, University of Milan Bicocca, Milan, Italy, for providing the macro SAS; and Ms. Judith Baggott for English editing.*  Costanzo S, Di Castelnuovo A, Donati MB, **Iacoviello L**, de Gaetano G. Cardiovascular and Overall Mortality Risk in Relation to Alcohol Consumption in Patients With Cardiovascular Disease. Circulation. 2010;121(17):1951-9.  ***This study was funded in part by a grant from the*** ***European Research Advisory Board (No. EA0827).***  di Giuseppe R, de Lorgeril M, Salen P, Laporte F, Di Castelnuovo A, Krogh V, Siani, A. Arnout, J. Cappuccio, F. P. van Dongen, M. Donati, M. B. de Gaetano, G. **Iacoviello, L.** Alcohol consumption and n-3 polyunsaturated fatty acids in healthy men and women from 3 European populations. American Journal of Clinical Nutrition. 2009;89(1):354-62.  ***Supported by the*** ***European Research Advisory Board (grant EA 05 20)*** *and the European Union (grant QLK1-2000-00100). MIUR (Ministero dell'Istruzione, Universita e Ricerca, Italia) Programma Triennale di Ricerca (grant D. 1588), and the Fondazione Invernizzi are currently supporting GdG and his associates at the Catholic University in Campobasso, Italy.*  Latella MC, Di Castelnuovo A, de Lorgeril M, Arnout J, Cappuccio FP, Krogh V, Siani, A. van Dongen, M. Donati, M. B. de Gaetano, G. **Iacoviello,** L. Genetic variation of alcohol dehydrogenase type 1C (ADH1C), alcohol consumption, and metabolic cardiovascular risk factors: Results from the IMMIDIET study. Atherosclerosis. 2009;207(1):284-90**.**  ***The IMMIDIET study was supported by*** *the European Union grant no. QLK1-2000-00100. MIUR (Ministero dell'Universita e Ricerca, Italia)-Programma Triennale di Ricerca ( grant D. 1588)* ***ERAB ( grant EA 05 20)*** *and Fondazione Invernizzi supported L. I. and her associates at the Catholic University in Campobasso, Italy.*  de Gaetano G, Costanzo S, Di Castelnuovo A, Badimon L, Bejko D, Alkerwi A, Chiva-Blanch, G. Estruch, R. La Vecchia, C. Panico, S. Pounis, G. Sofi, F. Stranges, S. Trevisan, M. Ursini, F. Cerletti, C. Donati, M. B. **Iacoviello, L.** Effects of moderate beer consumption on health and disease: A consensus document. Nutrition Metabolism and Cardiovascular Diseases. 2016;26(6):443-67***.*** ***Giovanni de Gaetano*** *is a consultant to the Web Newsletter of* ***Assobirra****, the Italian Association of the Beer and Malt Industries; Simona Costanzo: none; Augusto Di Castelnuovo: none; Ala’a Alkerwi: none;* ***Lina Badimon*** *is a Member of the Advisory Board of* ***Fundación Cerveza y Salud****; Dritan Bejko: none; Gemma Chiva-Blanch: none;* ***Ramon Estruch*** *is serving on the board of and receiving lecture fees from the* ***Research Foundation on Wine and Nutrition (FIVIN);*** *serving on the boards of the* ***Beer and Health Foundation and the European Foundation for Alcohol Research (ERAB)****; receiving lecture fees from* ***Cerveceros de España****; Carlo La Vecchia: none; Salvatore Panico: none; George Pounis: none; Francesco Sofi: none; Saverio Stranges: none; Maurizio Trevisan: none;* ***Fulvio Ursini*** *is a consultant to the Web Newsletter of* ***Assobirra****, the Italian Association of the Beer and Malt Industries; Chiara Cerletti: none; Maria Benedetta Donati: none; Licia Iacoviello: none.* ***This study was supported in part by Assobirra, the Italian Association of the Beer and Malt Industries.*** *This funding source had no involvement in either study design, or selection of the Panel members, or collection and interpretation of data, or the writing of the report nor in Panel’s decision to submit the manuscript for publication. We disclaim any other relationships with any industry or individuals who that might pose a conflict of interest in connection with the submitted article. The manuscript has been read and approved for submission to Nutrition, Metabolism and Cardiovascular Disease by all Authors.* |
|  | **Other declarations of alcohol industry support** | None identified |
|  | **Declarations by co-authors** | Sluik D, Jankovic N, Hughes M, O'Doherty MG, Schottker B, Drygas W, Rolandsson, O. Maennistoe, S. Ordonez-Mena, J. M. Ferrieres, J. Bamia, C. De Gaetano, G. Kiefte-De Jong, J. C. Franco, O. H. Sluijs, I. Spijkerman, A. M. W. Sans, S. Eriksson, S. Kromhout, D. Trichopoulou, A. Wilsgaard, T. Brenner, H. Kuulasmaa, K. Laatikainen, T. Soederberg, **S. Iacoviello,** L. Boffetta, P. Kee, F. Feskens, E. J. M.. Alcoholic beverage preference and diabetes incidence across Europe: the Consortium on Health and Ageing Network of Cohorts in Europe and the United States (CHANCES) project. European Journal of Clinical Nutrition. 2017;71(5):659-68.  *This analysis was part of the Consortium on Health and Ageing (CHANCES) project funded in the FP7 framework programme of the Directorate-General for Research and Innovation in the European Commission (grant 242244). The CHANCES project is coordinated by the Hellenic Health Foundation, Greece. Harmonization of the data from the MORGAM cohorts was also supported by European Union FP 7 project BiomarCaRE (278913).* ***The research of D Sluik was supported by the*** ***Dutch Beer Institute and the European Foundation for Alcohol Research (ERAB).*** |
| Maria Benedetta Donati | **Alcohol industry research funding declaration** | Costanzo S, Di Castelnuovo A, **Donati MB,** Iacoviello L, de Gaetano G. Wine, beer or spirit drinking in relation to fatal and non-fatal cardiovascular events: a meta-analysis. Eur J Epidemiol. 2011;26(11):833-50.  ***Supported in part by Cervisia Consulenze*** *and Istituto Nazionale per la Comunicazione. This was an investigator-initiated study. The partial sponsor of the study had no role in the selection of articles or conduct of the analyses or drafting of the manuscript. We disclaim any other relationships with industry that might pose a conflict of interest in connection with the submitted article.*  Costanzo S, Di Castelnuovo A, **Donati MB,** Iacoviello L, de Gaetano G. Alcohol Consumption and Mortality in Patients With Cardiovascular Disease A Meta-Analysis. Journal of the American College of Cardiology. 2010;55(13):1339-47.  *From the Laboratory of Genetic and Environmental Epidemiology, "RE ARTU" Research Laboratories, "John Paul II" Centre for High Technology Research and Education in Biomedical Sciences, Catholic University, Campobasso, Italy.* ***Supported in part by grant EA0827 from the*** ***European Research Advisory Board (ERAB).*** *The authors thank Professor Jozef Vermylen, Catholic University, Leuven, Belgium, for his critical review of the manuscript; Dr. Vincenzo Bagnardi, University of Milan Bicocca, Milan, Italy, for providing the macro SAS; and Ms. Judith Baggott for English editing.*  Costanzo S, De Curtis A, di Niro V, Olivieri M, Morena M, De Filippo CM,  Caradonna, E. Krogh, V. Serafini, M. Pellegrini, N. **Donati, M. B.** de Gaetano, G. Iacoviello, L. Postoperative atrial fibrillation and total dietary antioxidant capacity in patients undergoing cardiac surgery: The Polyphemus Observational Study. Journal of Thoracic and Cardiovascular Surgery. 2015;149(4):1175-82.  ***This study was supported by*** *the Italian Ministry of Health, Young Researchers Grant Number 2008-1146478,* ***The European Foundation for Alcohol Research (ERAB) Grant EA082****, The* ***International Organisation of Vine and Wine (OIV)*** ***Grant 2011 to S.C****., and the Project "Malattie cardiovascolari: ruoli di fattori genetici, acquisiti, nuovi approcci terapeutici e condizioni organizzative ottimali per la produzione delle conoscenze'' (D. MIUR n. 328 del 01/07/2010). These funding sources had no involvement in the study design; the collection, analysis, and interpretation of data; the writing of the article; or the authors' decision to submit the article for publication.*  Costanzo S, Di Castelnuovo A, **Donati MB,** Iacoviello L, de Gaetano G. Alcohol Consumption and Mortality in Patients With Cardiovascular Disease A Meta-Analysis. Journal of the American College of Cardiology. 2010;55(13):1339-47.  *From the Laboratory of Genetic and Environmental Epidemiology, "RE ARTU" Research Laboratories, "John Paul II" Centre for High Technology Research and Education in Biomedical Sciences, Catholic University, Campobasso, Italy.* ***Supported in part by grant EA0827 from the*** ***European Research Advisory Board (ERAB).*** *The authors thank Professor Jozef Vermylen, Catholic University, Leuven, Belgium, for his critical review of the manuscript; Dr. Vincenzo Bagnardi, University of Milan Bicocca, Milan, Italy, for providing the macro SAS; and Ms. Judith Baggott for English editing.*  Costanzo S, Di Castelnuovo A, **Donati MB,** Iacoviello L, de Gaetano G. Cardiovascular and Overall Mortality Risk in Relation to Alcohol Consumption in Patients With Cardiovascular Disease. Circulation. 2010;121(17):1951-9.  ***This study was funded in part by a grant from the European Research Advisory Board (No. EA0827).***  di Giuseppe R, de Lorgeril M, Salen P, Laporte F, Di Castelnuovo A, Krogh V, Krogh V, Siani, A. Arnout, J. Cappuccio, F. P. van Dongen, M. **Donati, M. B.** de Gaetano, G. Iacoviello, L. Alcohol consumption and n-3 polyunsaturated fatty acids in healthy men and women from 3 European populations. American Journal of Clinical Nutrition. 2009;89(1):354-62.  ***Supported by the European Research Advisory Board (grant EA 05 20)*** *and the European Union (grant QLK1-2000-00100). MIUR (Ministero dell'Istruzione, Universita e Ricerca, Italia) Programma Triennale di Ricerca (grant D. 1588), and the Fondazione Invernizzi are currently supporting GdG and his associates at the Catholic University in Campobasso, Italy.*  Latella MC, Di Castelnuovo A, de Lorgeril M, Arnout J, Cappuccio FP, Krogh V, Siani, A. van Dongen, M. **Donati, M. B.** de Gaetano, G. Iacoviello, L. Genetic variation of alcohol dehydrogenase type 1C (ADH1C), alcohol consumption, and metabolic cardiovascular risk factors: Results from the IMMIDIET study. Atherosclerosis. 2009;207(1):284-90.  ***The IMMIDIET study was supported by*** *the European Union grant no. QLK1-2000-00100. MIUR (Ministero dell'Universita e Ricerca, Italia)-Programma Triennale di Ricerca ( grant D. 1588)* ***ERAB ( grant EA 05 20)*** *and Fondazione Invernizzi supported L. I. and her associates at the Catholic University in Campobasso, Italy.*  de Gaetano G, Costanzo S, Di Castelnuovo A, Badimon L, Bejko D, Alkerwi A, Chiva-Blanch, G. Estruch, R. La Vecchia, C. Panico, S. Pounis, G. Sofi, F. Stranges, S. Trevisan, M. Ursini, F. Cerletti, C. **Donati, M. B**. Iacoviello, L. Effects of moderate beer consumption on health and disease: A consensus document. Nutrition Metabolism and Cardiovascular Diseases. 2016;26(6):443-67. ***Giovanni de Gaetano*** *is a consultant to the Web Newsletter of* ***Assobirra****, the Italian Association of the Beer and Malt Industries; Simona Costanzo: none; Augusto Di Castelnuovo: none; Ala’a Alkerwi: none;* ***Lina Badimon*** *is a Member of the Advisory Board of* ***Fundación Cerveza y Salud****; Dritan Bejko: none; Gemma Chiva-Blanch: none;* ***Ramon Estruch*** *is serving on the board of and receiving lecture fees from the* ***Research Foundation on Wine and Nutrition (FIVIN);*** *serving on the boards of the* ***Beer and Health Foundation and the European Foundation for Alcohol Research (ERAB)****; receiving lecture fees from* ***Cerveceros de España****; Carlo La Vecchia: none; Salvatore Panico: none; George Pounis: none; Francesco Sofi: none; Saverio Stranges: none; Maurizio Trevisan: none;* ***Fulvio Ursini*** *is a consultant to the Web Newsletter of* ***Assobirra****, the Italian Association of the Beer and Malt Industries; Chiara Cerletti: none; Maria Benedetta Donati: none; Licia Iacoviello: none.* ***This study was supported in part by Assobirra, the Italian Association of the Beer and Malt Industries.*** *This funding source had no involvement in either study design, or selection of the Panel members, or collection and interpretation of data, or the writing of the report nor in Panel’s decision to submit the manuscript for publication. We disclaim any other relationships with any industry or individuals who that might pose a conflict of interest in connection with the submitted article. The manuscript has been read and approved for submission to Nutrition, Metabolism and Cardiovascular Disease by all Authors.* |
|  | **Other declarations of alcohol industry support** | None identified |
|  | **Declarations by co-authors** | None identified |
| Carlo La Vecchia | **Alcohol industry research funding declaration** | de Gaetano G, Costanzo S, Di Castelnuovo A, Badimon L, Bejko D, Alkerwi A, Chiva-Blanch, G. Estruch, R. **La Vecchia,** C. Panico, S. Pounis, G. Sofi, F. Stranges, S. Trevisan, M. Ursini, F. Cerletti, C. Donati, M. B. Iacoviello, L. Effects of moderate beer consumption on health and disease: A consensus document. Nutrition Metabolism and Cardiovascular Diseases. 2016;26(6):443-67.  ***Giovanni de Gaetano*** *is a consultant to the Web Newsletter of* ***Assobirra****, the Italian Association of the Beer and Malt Industries; Simona Costanzo: none; Augusto Di Castelnuovo: none; Ala’a Alkerwi: none; Lina Badimon is a Member of the Advisory Board of Fundación Cerveza y Salud; Dritan Bejko: none; Gemma Chiva-Blanch: none;* ***Ramon Estruch*** *is serving on the board of and receiving lecture fees from the* ***Research Foundation on Wine and Nutrition (FIVIN);*** *serving on the boards of the* ***Beer and Health Foundation*** *and the* ***European Foundation for Alcohol Research (ERAB);*** *receiving lecture fees from* ***Cerveceros de España****; Carlo La Vecchia: none; Salvatore Panico: none; George Pounis: none; Francesco Sofi: none; Saverio Stranges: none; Maurizio Trevisan: none;* ***Fulvio Ursini*** *is a consultant to the Web Newsletter of* ***Assobirra****, the Italian Association of the Beer and Malt Industries; Chiara Cerletti: none; Maria Benedetta Donati: none; Licia Iacoviello: none.* ***This study was supported in part by Assobirra, the Italian Association of the Beer and Malt Industries.*** *This funding source had no involvement in either study design, or selection of the Panel members, or collection and interpretation of data, or the writing of the report nor in Panel’s decision to submit the manuscript for publication. We disclaim any other relationships with any industry or individuals who that might pose a conflict of interest in connection with the submitted article. The manuscript has been read and approved for submission to Nutrition, Metabolism and Cardiovascular Disease by all Authors*. |
|  | **Other declarations of alcohol industry support** | None identified |
|  | **Declarations by co-authors** | None identified |
| Antonella Zambon | **Alcohol industry research funding declaration** | Giovanni Addolorato, Lorenzo Leggio, Anna Ferrulli, Silvia Cardone, Luisa Vonghia, Antonio Mirijello, Ludovico Abenavoli, Cristina D'Angelo, Fabio Caputo, **Antonella Zambon,** Paul S Haber, Giovanni Gasbarrini. Effectiveness and safety of baclofen for maintenance of alcohol abstinence in alcohol-dependent patients with liver cirrhosis: randomised, double-blind controlled study. The Lancet, Volume 370, Issue 9603, 2007: 1915-1922,  ***This study was supported by*** *the Italian Ministry for University, Scientific and Technological Research (MURST), and by the* ***European Research Advisory Board (ERAB).*** |
|  | **Other declarations of alcohol industry support** | None identified |
|  | **Declarations by co-authors** | Leggio L, Ferrulli A, **Zambon A,** Caputo F, Kenna GA, Swift RM, Addolorato, G. Baclofen promotes alcohol abstinence in alcohol dependent cirrhotic patients with hepatitis C virus (HCV) infection. Addictive Behaviors. 2012;37(4):561-4.  ***Funding for this study was partially provided by a grant (L.L. and G.A.) and by an Exchange Award (L.L.), both from the*** ***European Foundation for Alcohol Research (ERAB)****; and a grant (G.A.) from the Italian Ministry for University, Scientific and Technological Research (MURST). Both* ***ERAB*** *and MURST had no further role in study design; in the collection, analysis and interpretation of data; in the writing of the report; and in the decision to submit the paper for publication.* |
| **SUBNETWORK 3 (Contained 5 systematic reviews)** | | |
| Kenneth J. Mukamal | **Alcohol industry research funding declaration** | **Mukamal KJ.** A safe level of alcohol consumption: the right answer demands the right question. J Intern Med.10**.**  ***Dr. Mukamal was the principal investigator of a trial funded by the US National Institutes of Health that received contributions to the Foundation for NIH from the alcoholic beverage industry* in 2016-18****. Dr. Mukamal grateful to Drs. Kenneth Warren and Eric Rimm for helpful suggestions.*This is referring to the MACH trial of which the funders were* ***Anheuser-Busch InBev, Carlsberg Breweries A/S, Diageo plc, Heineken, Pernod Ricard USA LLC****..* [*https://clinicaltrials.gov/ct2/show/NCT03169530*](https://clinicaltrials.gov/ct2/show/NCT03169530) |
|  | **Other declarations of alcohol industry support** | Collins MA, Neafsey EJ, **Mukamal KJ,** Gray MO, Parks DA, Das DK, Korthuis, R. J.. Alcohol in Moderation, Cardioprotection, and Neuroprotection: Epidemiological Considerations and Mechanistic Studies. Alcoholism-Clinical and Experimental Research. 2009;33(2):206-19.  ***The roundtable was supported in part by*** *the Loyola University Neuroscience Institute and the* ***Alcohol Beverage Medical Research Foundation (ABMRF****). This article summarizes the proceedings of a roundtable organized/chaired by Dr. Michael A. Collins and* ***co‐chaired by*** ***Dr. Kenneth J. Mukamal*** *that was held at the Research Society on Alcoholism meetings in Chicago, Illinois, July 7–11, 2007. Abstracts are included in Alcohol Clin Exp Res 2007:31(Suppl.6), 301A–302A.*  Spiegelman D, Lovato LC, Khudyakov P, Wilkens TL, Adebamowo CA, Adebamowo SN, Appel LJ, Beulens JW, Coughlin JW, Dragsted LO, Edenberg HJ, Eriksen JN, Estruch R, Grobbee DE, Gulayin PE, Irazola V, Krystal JH, Lazo M, Murray MM, Rimm EB, Schrieks IC, Williamson JD, **Mukamal KJ.** The Moderate Alcohol and Cardiovascular Health Trial (MACH15): Design and methods for a randomized trial of moderate alcohol consumption and cardiometabolic risk. Eur J Prev Cardiol. 2020  *The author(s) declared the following potential conflicts of interest with respect to research, authorship, and/or publication of this article:* ***LOD, JNE, and TLW*** *received a Semper Ardens grant from the* ***Carlsberg Foundation*** *running 2015– 2021. The funder had no role in any phases of the study conduction or in the decision to prepare or publish the manuscript.* ***LOD*** *also reports funding from the Danish Innovation Foundation (governmental). RE reports grants from the Spanish Institute of Health “Carlos III”; Ministry of Agriculture, Fisheries and Food, Government of Spain; Autonomic Government of Catalonia, Spain; University of Barcelona, Spain;* ***Cerveza y Salud, Spain****; Fundacion Dieta Mediterranea, Spain; European Union, Brussels; NIAAA, USA. Additionally, RE received personal fees for given lectures from* ***Brewers of Europe, Belgium; Fundacion Cerveza y Salud, Spain; Pernaud-Ricard, Mexico;*** *Instituto Cervantes from Albuquerque/USA, Milan/Italy, and Tokyo/Japan; Lilly Laboratories and Uriach Laboratories, Spain; and the* ***Wine and Culinary International Forum****, Spain.* ***JHK and KJM attended meetings funded by NIAAA and private-sector members of the*** ***alcohol industry*** *in 2013–2014 prior to any application for or receipt of grant funding for the trial.* ***MMM*** *has worked for NIAAA and has also received reimbursement for travel from the* ***International Alliance for Responsible Drinking****.* ***ISC*** *reports funding between 2011 and 2015 from the Dutch Ministry of Economic Affairs, Agriculture and Innovation; the* ***Dutch Foundation for Alcohol Research (SAR)****; and the Netherlands Organization for Applied Scientific Research. Their joint aim was to independently study the health effects of moderate alcohol consumption. The funders had no role in study design, data collection and analysis, decision to publish, or preparation of the manuscripts during that period. The remaining authors DS, LCL, PK, CAA, SNA, LJA, JWJB, JWC, HJE, DDG, PEG, WI, ML, EBR, and JDW declare no conflicts of interest.* |
|  | **Declarations by co-authors** | Beulens JWJ, Rimm EB, Hu FB, Hendriks HFJ, **Mukamal KJ.** Alcohol Consumption, Mediating, Biomarkers, and Risk of Type 2 Diabetes Among Middle-Aged Women. Diabetes Care. 2008;31(10):2050-5.  *This work was supported by National Institutes of Health grants R01AA011181 and DK58845, a travel grant from the Dutch Heart Association,* ***and a research exchange award from European Research Advisory Board (J.W.J.B.).***  Koch M, Costanzo S, Fitzpatrick AL, Lopez OL, DeKosky S, Kuller LH, Price, J. Mackey, R. H. Jensen, M. K. **Mukamal, K. J.** Alcohol Consumption, Brain Amyloid-beta Deposition, and Brain Structural Integrity Among Older Adults Free of Dementia. J Alzheimers Dis. 2020;74(2):509-19.  *Samples from the National Cell Repository for Alzheimer's Disease (NCRAD), which receives government support under a cooperative agreement grant (U24 AG21886) awarded by the National Institute on Aging (NIA), were used in this study. This work was supported by the NIH/NINDS (1R01NS089638-01A1).* ***Simona Costanzo is principal investigator of an ongoing study supported by a research grant from the*** ***European Foundation for Alcohol Research (ERAB, EA1767).*** *The funding sources had no role in study design; in the collection, analysis and interpretation of data; in the writing of the report; and in the decision to submit the article for publication.*  **Mukamal, K.J.,** Clowry, C.M., Murray, M.M., Hendriks, H.F., Rimm, E.B., Sink, K.M., Adebamowo, C.A., Dragsted, L.O., Lapinski, P.S., Lazo, M. and Krystal, J.H. (2016), Moderate Alcohol Consumption and Chronic Disease: The Case for a Long‐Term Trial. Alcohol Clin Exp Res, 40: 2283-2291.  *This work was supported by grants U13AA023452, U34AA023258, and U10AA025286 from the National Institute on Alcohol Abuse and Alcoholism.* ***Drs. Hendriks and Dragsted*** *have conducted short‐term feeding studies with alcohol at institutions (TNO, University of Copenhagen) that have* ***received partial support from members of the alcohol industry****. Dr. Krystal has served on the advisory boards and/or holds stock in several companies with interests in psychiatric disorders.*  **Mukamal K,** Lazo M. Alcohol and cardiovascular disease. Bmj-British Medical Journal. 2017;356:2.  *We have read and understood the BMJ Group policy on declaration of interests and declare the following interests:* ***ML previously held a research grant from*** ***ABMRF/The Foundation for Alcohol Research****, which is funded in part by industry, for a project on alcohol and liver disease.*  Schrieks IC, Heil ALJ, Hendriks HFJ, **Mukamal KJ,** Beulens JWJ. The Effect of Alcohol Consumption on Insulin Sensitivity and Glycemic Status: A Systematic Review and Meta-analysis of Intervention Studies. Diabetes Care. 2015;38(4):723-32.  ***I.C.S. and H.F.J.H. were supported by*** *both the Dutch Ministry of Economic Affairs, Agriculture and Innovation and the* ***Dutch Foundation for Alcohol Research****, representing Dutch producers of and traders in beer, wine, and spirits and The Netherlands Organization for Applied Scientific Research. Their joint aim is to independently study the health effects of moderate alcohol consumption.*  Schrieks IC, Wei MY, Rimm EB, Okereke OI, Kawachi I, Hendriks HFJ**,** **Mukamal, K.** Bidirectional associations between alcohol consumption and health-related quality of life amongst young and middle-aged women. J Intern Med. 2016;279(4):376-87.  *This work was supported by the Nurses' Health Study II grant UM1 CA176726 from the National Institutes of Health.* ***ICS and HFJH were supported*** *both by the Dutch Ministry of Economic Affairs, Agriculture and Innovation and* ***by the*** ***Dutch Foundation for Alcohol Research (SAR)*** *representing Dutch producers of and traders in beer, wine and spirits and The Netherlands Organization for Applied Scientific Research (TNO) (Grant EZ1503). Their joint aim is to independently investigate the health effects of moderate alcohol consumption. The funding sources had no role in conducting, analysing, or interpreting the study results or in the decision to submit the manuscript for publication.* |
| Eric B Rimm | **Alcohol industry research funding declaration** | **Rimm EB,** Klatsky A, Grobbee D, Stampfer MJ. Review of moderate alcohol consumption and reduced risk of coronary heart disease: Is the effect due to beer, wine, or spirits? Br Med J. 1996;312(7033):731-6.  ***This report was funded by the International Life Sciences Institute (ILSI Europe Alcohol Task Force).*** |
|  | **Other declarations of alcohol industry support** | **Rimm EB,** Williams P, Fosher K, Criqui M, Stampfer MJ. Moderate alcohol intake and lower risk of coronary heart disease: meta-analysis of effects on lipids and haemostatic factors. Br Med J. 1999;319(7224):1523-8D.  *Funding:* ***The Europe Alcohol Task Force of the International Life Sciences Institute****. Competing interests:* ***EBR*** *has received both honorariums for speaking at academic conferences and travel expenses from* ***alcohol related organisations****.* |
|  | **Declarations by co-authors** | Beulens JWJ, **Rimm EB,** Hu FB, Hendriks HFJ, Mukamal KJ. Alcohol Consumption, Mediating, Biomarkers, and Risk of Type 2 Diabetes Among Middle-Aged Women. Diabetes Care. 2008;31(10):2050-5.  *This work was supported by National Institutes of Health grants R01AA011181 and DK58845, a travel grant from the Dutch Heart Association, and* ***a research exchange award from European Research Advisory Board (J.W.J.B.).*** |
| Michael Criqui | **Alcohol industry research funding declaration** | Rimm EB, Williams P, Fosher K, **Criqui M,** Stampfer MJ. Moderate alcohol intake and lower risk of coronary heart disease: meta-analysis of effects on lipids and haemostatic factors. Br Med J. 1999;319(7224):1523-8D.  *Funding:* ***The Europe Alcohol Task Force of the International Life Sciences Institute****.* |
|  | **Other declarations of alcohol industry support** | None identified |
|  | **Declarations by co-authors** | Vos T, Barber RM, Bell B, Bertozzi-Villa A, Biryukov S, Bolliger I, Charlson, F. Davis, A. Degenhardt, L. Dicker, D. Duan, L. Erskine, H. Feigin, V. L. Ferrari, A. J. Fitzmaurice, C. Fleming, T. Graetz, N. Guinovart, C. Haagsma, J. et al. Global, regional, and national incidence, prevalence, and years lived with disability for 301 acute and chronic diseases and injuries in 188 countries, 1990-2013: a systematic analysis for the Global Burden of Disease Study 2013. Lancet. 2015;386(9995):743-800.  ***DS*** *has received research grants or consultancy honoraria from Abbott,* ***ABMRF****.* |
| Arthur Klatsky | **Alcohol industry research funding declaration** | Rimm EB, **Klatsky A,** Grobbee D, Stampfer MJ. Review of moderate alcohol consumption and reduced risk of coronary heart disease: Is the effect due to beer, wine, or spirits? Br Med J. 1996;312(7033):731-6.  ***This report was funded by the International Life Sciences Institute (ILSI Europe Alcohol Task Force).***  **Klatsky AL,** Gunderson E. Alcohol and hypertension: a review. J Am Soc Hypertens. 2008;2(5):307-17.  ***Portions of the material in this article include research supported by the*** ***Alcoholic Beverage Medical Research Foundation****, Inc, Baltimore, Maryland, the Community Service Program of The Kaiser Permanente Medical Care Program, and by the Robert Wood Johnson Foundation.*  **Klatsky AL,** Li Y, Baer D, Armstrong MA, Udaltsova N, Friedman GD. Alcohol Consumption and Risk of Hematologic Malignancies. Ann Epidemiol. 2009;19(10):746-53.  *This Study was funded by a research grant from the Kaiser Foundation Research Institute.* ***Data collection from 1978 to 1985 was supported by a grant from the*** ***Alcoholic Beverage Medical Research Foundation****, Baltimore*  Li Y, Baer D, Friedman GD, Udaltsova N, Shim V, **Klatsky AL.** Wine, liquor, beer and risk of breast cancer in a large population. Eur J Cancer. 2009;45(5):843-50.  *This study was supported by a grant from the Kaiser Foundation Research Institute.* ***Data collection from 1978 to 1985 were supported by a grant from the Alcoholic Beverage Medical Research Foundation****, Baltimore, MD.*  Tran HN, Siu S, Iribarren C, Udaltsova N**, Klatsky AL.** Ethnicity and Risk of Hospitalization for Asthma and Chronic Obstructive Pulmonary Disease. Ann Epidemiol. 2011;21(8):615-22.  *All analyses were performed in the period 2007-2010 with the support of a community budget grant from the Kaiser Foundation Research Institute with Dr. Stanton Siu as principal investigator (PI).* ***Data collection from 1978 through 1985 was supported by a grant from the Alcoholic Beverage Medical Research Foundation, Baltimore, MD, with Dr. Arthur Klatsky as PI.*** *The investigators and authors had complete freedom to decide about analyses and to choose material to be included in the manuscript and none has any conflict of interest.*  **Klatsky AL,** Udaltsova N, Li Y, Baer D, Tran HN, Friedman GD. Moderate alcohol intake and cancer: the role of underreporting. Cancer Causes Control. 2014;25(6):693-9.  *The research was performed at the Division of Research of the Kaiser Permanente Medical Care Program, Oakland CA with support by a Community Budget grant from the Kaiser Foundation Research Institute to Yan Li, MD, PhD as Principal Investigator.* ***Data collection in 1978-1985 was supported by a grant to Dr. Arthur L. Klatsky from the Alcoholic Beverage Medical Research Foundation*** *of Baltimore, MD. We are grateful to Cynthia Landy for assistance with data collection.*  Tran HN, Li Y, Udaltsova N, Armstrong MA, Friedman GD, **Klatsky AL.** Risk of cancer in Asian Americans: a Kaiser Permanente cohort study. Cancer Causes Control. 2016;27(10):1197-207. *The research was performed at the Division of Research of the Kaiser Permanente Medical Care Program, Oakland, CA, with support by a Community Budget grant from the Kaiser Foundation Research Institute to Yan Li, MD, PhD as Principal Investigator.* ***Data collection in 1978-1985 was supported by a grant to Dr. Arthur L. Klatsky from the Alcoholic Beverage Medical Research Foundation*** *of Baltimore, MD. We are grateful to Cynthia Landy for assistance with data collection.*  **Klatsky, A.L.** and Udaltsova, N. (2013), Commentaries. Addiction, 108: 1549-1552.  *This work was supported by a grant from the Community Budget program of the Kaiser Permanente Medical Care Program in Oakland, CA, USA.* ***Data collection in 1978–85 was supported by a grant from the Alcoholic Beverage Medical Research Foundation****. No funding source played any oversight role in the analysis or in the preparation of the manuscript.* ***Dr Klatsky*** ***is a member of the International Scientific Forum on Alcohol Research (ISFAR) and on the editorial board of Alcohol in Moderation (AIM), both of which are associated with the alcohol industry.*** *Neither of these entities has supplied him or his institution with any financial support.* |
|  | **Other declarations of alcohol industry support** | None identified |
|  | **Declarations by co-authors** | None identified |
| Diederick Grobbee | **Alcohol industry research funding declaration** | Rimm EB, Klatsky A, **Grobbee D,** Stampfer MJ. Review of moderate alcohol consumption and reduced risk of coronary heart disease: Is the effect due to beer, wine, or spirits? Br Med J. 1996;312(7033):731-6.  ***This report was funded by the International Life Sciences Institute (ILSI Europe Alcohol Task Force).*** |
|  | **Other declarations of alcohol industry support** | None identified |
|  | **Declarations by co-authors** | None identified |
| Meir J Stampfer | **Alcohol industry research funding declaration** | Rimm EB, Klatsky A, Grobbee D, **Stampfer MJ.** Review of moderate alcohol consumption and reduced risk of coronary heart disease: Is the effect due to beer, wine, or spirits? Br Med J. 1996;312(7033):731-6.  ***This report was funded by the*** ***International Life Sciences Institute (ILSI Europe Alcohol Task Force).***  Rimm EB, Williams P, Fosher K, Criqui M, **Stampfer MJ.** Moderate alcohol intake and lower risk of coronary heart disease: meta-analysis of effects on lipids and haemostatic factors. Br Med J. 1999;319(7224):1523-8D.  ***Funding: The Europe Alcohol Task Force of the International Life Sciences Institute.*** |
|  | **Other declarations of alcohol industry support** | None identified |
|  | **Declarations by co-authors** | None identified |
| Paige Williams | **Alcohol industry research funding declaration** | Rimm EB, **Williams P,** Fosher K, Criqui M, Stampfer MJ. Moderate alcohol intake and lower risk of coronary heart disease: meta-analysis of effects on lipids and haemostatic factors. Br Med J. 1999;319(7224):1523-8D.  ***Funding:*** ***The Europe Alcohol Task Force of the International Life Sciences Institute****.* |
|  | **Other declarations of alcohol industry support** | None identified |
|  | **Declarations by co-authors** | None identified |
| Kerry Fosher | **Alcohol industry research funding declaration** | Rimm EB, Williams P, **Fosher K,** Criqui M, Stampfer MJ. Moderate alcohol intake and lower risk of coronary heart disease: meta-analysis of effects on lipids and haemostatic factors. Br Med J. 1999;319(7224):1523-8D.  ***Funding: The Europe Alcohol Task Force of the International Life Sciences Institute.*** |
|  | **Other declarations of alcohol industry support** | None identified |
|  | **Declarations by co-authors** | None identified |
| **SUBNETWORK 5 (Contained 3 systematic reviews)** | | |
| Martin McKee | **Alcohol industry research funding declaration** | None identified |
|  | **Other declarations of alcohol industry support** | None identified |
|  | **Declarations by co-authors** | Vos T, Barber RM, Bell B, Bertozzi-Villa A, Biryukov S, Bolliger I, Charlson, F. Davis, A. Degenhardt, L. Dicker, D. Duan, L. Erskine, H. Feigin, V. L. Ferrari, A. J. Fitzmaurice, C. Fleming, T. Graetz, N. Guinovart, C. Haagsma, J. et al. Global, regional, and national incidence, prevalence, and years lived with disability for 301 acute and chronic diseases and injuries in 188 countries, 1990-2013: a systematic analysis for the Global Burden of Disease Study 2013. Lancet. 2015;386(9995):743-800.  ***DS has received research grants or consultancy honoraria from Abbott, ABMRF*.** |
| **SUBNETWORK 6 (Contained 2 systematic reviews)** | | |
| Tanya Chikritzhs | **Alcohol industry research funding declaration** | None identified |
|  | **Other declarations of alcohol industry support** | None identified |
|  | **Declarations by co-authors** | Graham K, Miller P, **Chikritzhs T,** Bellis MA, Clapp JD, Hughes K, Toomey, T. L. Wells, S. Reducing intoxication among bar patrons: some lessons from prevention of drinking and driving. Addiction. 2014;109(5):693-8**.**  *During the past 5 years, the Centre for Public Health (****Bellis, Hughes****)* ***has received a grant from Drinkaware*** ***to undertake an independent study of drinking behaviours among students and MAB has provided them with independent medical advice****.* ***Drinkaware*** *is an independent UK-wide charity supported by voluntary contributions from the alcohol and supermarket industries and governed through a memorandum of understanding between the Department of Health, Home Office, Scottish Executive, Welsh Assembly Government, Northern Ireland Office and Portman Group.*  Stockwell T, **Chikritzhs T,** Naimi T, Zhao JH. ISFAR Doth Protest Too Much: Another Attempt From Industry Sympathizers to Marginalize Scientific Skepticism About Alcohol's Hypothesized Health Benefits? J Stud Alcohol Drugs. 2016;77(5):839-41.  *Statement for Tim Stockwell: Ten years ago,* ***Tim Stockwell*** ***received expenses to attend two meetings from the then International Center on Alcohol Policy, an organization funded by alcohol industry groups.*** *Three years ago, he accepted funds from Lundbeck, a Danish pharmaceutical company, to attend a meeting to critique research on a drug they had developed for the treatment of alcohol dependence. He is currently contracted by the Swedish alcohol monopoly, Systembolaget, to conduct an international collaborative study concerning the policy impacts of government alcohol monopolies on health and safety.* |
| Tim Stockwell | **Alcohol industry research funding declaration** | None identified |
|  | **Other declarations of alcohol industry support** | **Stockwell T,** Chikritzhs T, Naimi T, Zhao JH. ISFAR Doth Protest Too Much: Another Attempt From Industry Sympathizers to Marginalize Scientific Skepticism About Alcohol's Hypothesized Health Benefits? J Stud Alcohol Drugs. 2016;77(5):839-41.  *Statement for Tim Stockwell: Ten years ago,* ***Tim Stockwell received expenses to attend two meetings from the then International Center on Alcohol Policy, an organization funded by alcohol industry groups.*** *Three years ago, he accepted funds from Lundbeck, a Danish pharmaceutical company, to attend a meeting to critique research on a drug they had developed for the treatment of alcohol dependence. He is currently contracted by the Swedish alcohol monopoly, Systembolaget, to conduct an international collaborative study concerning the policy impacts of government alcohol monopolies on health and safety.*  **Stockwell T,** Zhao J, Naimi T, Chikritzhs T. Response: Moderate Use of an “Intoxicating Carcinogen” Has No Net Mortality Benefit—Is This True and Why Does It Matter? J Stud Alcohol Drugs. 2016 Mar;77(2):205–7.  *Statement for* ***Tim Stockwell*** ***Ten years ago I received expenses from the then International Center on Alcohol Policy to attend meetings in New York and Belfast. This organization is funded by alcohol industry groups.*** *I no longer accept funds for any purposes from such sources. I am currently contracted by the Swedish alcohol monopoly, Systembolaget, to conduct an international collaborative study concerning the policy impacts of government alcohol monopolies on health and safety. Systembolaget was set up to limit the profit incentive in providing alcohol to the public and to minimize adverse health and safety consequences. Three years ago I accepted funds from Lundbeck, a Danish international pharmaceutical company, to attend a meeting to critique research on a drug they had developed for the treatment of alcohol dependence. I received a fee and travel expenses for a half-day meeting. More than 30 years ago I was paid by a grant from the German drug company Merck to conduct a study of a drug they were developing to treat alcohol dependence. This was paid to the Addiction Research Unit, Institute of Psychiatry, University of London, and paid my salary for 1 year.* |
|  | **Declarations by co-authors** | None identified |
| **SUBNETWORK 9 (Contained 2 systematic reviews)** | | |
| Lorraine. G. Ogden | **Alcohol industry research funding declaration** | None identified |
|  | **Other declarations of alcohol industry support** | None identified |
|  | **Declarations by co-authors** | **Ogden LG,** Stroebele N, Wyatt HR, Catenacci VA, Peters JC, Stuht J, Wing, R. R. Hill, J. O. Cluster Analysis of the National Weight Control Registry to Identify Distinct Subgroups Maintaining Successful Weight Loss. Obesity. 2012;20(10):2039-47.  *Partial funding was provided by The Procter & Gamble Company, Cincinnati, Ohio.;* ***J.O.H****. discloses the following: General Mills (Advisory Board); Coca-Cola (Consulting);* ***American Beverage Institute*** *(Research Support); Active Planet (Patent); Retrofit (Equity Stake).* ***H. R. W****. discloses the following: Wellspring (Consulting);* ***American Beverage Institute*** *(Research Support); Norvo Nordisc (Research Support); Active Planet (Patent); Up to Date (Royalties); Retrofit (Equity Stake). The other authors declared no conflict of interest.* |
| **SUBNETWORK 14 (Contained one review – i.e. isolated subnetwork)** | | |
| Romina de Lorgeril di Giuseppe | **Alcohol industry research funding declaration** | **di Giuseppe R,** de Lorgeril M, Salen P, Laporte F, Di Castelnuovo A, Krogh V, Siani, A. Arnout, J. Cappuccio, F. P. van Dongen, M. Donati, M. B. de Gaetano, G. Iacoviello, L. Alcohol consumption and n-3 polyunsaturated fatty acids in healthy men and women from 3 European populations. American Journal of Clinical Nutrition. 2009;89(1):354-62**.**  ***Supported by the*** ***European Research Advisory Board (grant EA 05 20)*** *and the European Union (grant QLK1-2000-00100). MIUR (Ministero dell'Istruzione, Universita e Ricerca, Italia) Programma Triennale di Ricerca (grant D. 1588), and the Fondazione Invernizzi are currently supporting GdG and his associates at the Catholic University in Campobasso, Italy*. |
|  | **Other declarations of alcohol industry support** | None identified |
|  | **Declarations by co-authors** | None identified |
| Heiner Boeing | **Alcohol industry research funding declaration** | Castello A, Pollan M, Buijsse B, Ruiz A, Casas AM, Baena-Canada JM, Lope, V. Antolin, S. Ramos, M. Munoz, M. Lluch, A. de Juan-Ferre, A. Jara, C. Jimeno, M. A. Rosado, P. Diaz, E. Guillem, V. Carrasco, E. Perez-Gomez, B. Vioque, J. **Boeing, H.** Martin, M. Geicam, Spanish Mediterranean diet and other dietary patterns and breast cancer risk: case-control EpiGEICAM study. Br J Cancer. 2014;111(7):1454-62.  ***This work was supported by*** *the Fundacion Cientifica Asociacion Espanola Contra el Cancer (AECC) (Scientific Foundation of the Spanish Association Against Cancer);* ***Fundacion Cerveza y Salud 2005 (Beer and Health Foundation 2005)****; Sociedad Espanola de Oncologia Medica (SEOM) (Spanish Society of Medical Oncology); Federacion de Mujeres con Cancer de Mama (FECMA) (Association of Women with Breast Cancer) and Fondo de Investigacion Sanitaria (FIS) (Health Research Fund).* |
|  | **Other declarations of alcohol industry support** | None identified |
|  | **Declarations by co-authors** | None identified |
| **SUBNETWORK 22 (Contained one review – i.e. isolated subnetwork)** | | |
| Lando L J Koppes | **Alcohol industry research funding declaration** | **Koppes, L**.L.J., Dekker, J.M., Hendriks, H.F.J. Bouter, L.M, Heine, R.J. Meta-analysis of the relationship between alcohol consumption and coronary heart disease and mortality in type 2 diabetic patients. Diabetologia 49, 648–652 (2006).  *The authors wish to thank I. Riphagen, Medical Library, Vrije University, Amsterdam, for her assistance with the literature search.* ***This work was supported by an unconditional grant from the Alcohol Task Force of the European branch of the International Life Sciences Institute (ILSI Europe). Industry members of this task force are Allied Domecq, Brasseries Kronenbourg, Diageo, Heineken and Moët et Chandon****. The opinions expressed in this article are those of the authors and do not necessarily represent the views of* ***ILSI Europe****.*  van Dijk CE, de Boer MR, **Koppes LLJ,** Roos JC, Lips P, Twisk JWR. Positive association between the course of vitamin D intake and bone mineral density at 36 years in men. Bone. 2009;44(3):437-41.  ***This work was supported by grants from*** *the Dairy Foundation on Nutrition and Health, the Dutch Heart Foundation (Grant 7605179051), the Dutch Prevention Fund (Grants 28-189a, 28-1106 and 281106-1), The Dutch Ministry of Health Welfare and Sports (Grand 90-170), the Dutch Olympic Committee/Netherlands Sport Federation,* ***Heineken*** *Inc., and the Scientific Board of Smoking and Health. We thank all participants of the AGAHLS.*  Veldhuis L, **Koppes LLJ,** Driessen MT, Samoocha D, Twisk JWR. Effects of dietary fibre intake during adolescence on the Subnetworks of the metabolic syndrome at the age of 36 years: the Amsterdam Growth and Health Longitudinal Study. J Hum Nutr Diet. 2010;23(6):601-8.  *All authors (LV, LK, MD, DS and JT) declare that they have no conflict of interest. The authors wish to thank the AGAHLS participants for investing their time and efforts.* ***The AGAHLS was financially supported by*** *Dutch Heart Foundation grants 76051-79051, Dutch Prevention Fund grants 28-189a, 28-1106 and 28-1106-1, Dutch Ministry of Well Being and Public Health grant 90-170, the Dairy Foundation on Nutrition and Health, the Dutch Olympic Committee/Netherlands Sports Federation,* ***Heineken*** *Inc. and the Scientific Board of Smoking and Health.* |
|  | **Other declarations of alcohol industry support** | None identified |
|  | **Declarations by co-authors** | None identified |
| Jacqueline M. Dekker | **Alcohol industry research funding declaration** | Koppes, L.L.J., **Dekker,** J.M., Hendriks, H.F.J. Bouter, L.M, Heine, R.J. Meta-analysis of the relationship between alcohol consumption and coronary heart disease and mortality in type 2 diabetic patients. Diabetologia 49, 648–652 (2006).  *The authors wish to thank I. Riphagen, Medical Library, Vrije University, Amsterdam, for her assistance with the literature search.* ***This work was supported by an unconditional grant from the Alcohol Task Force of the European branch of the International Life Sciences Institute (ILSI Europe). Industry members of this task force are Allied Domecq, Brasseries Kronenbourg, Diageo, Heineken and Moët et Chandon.*** *The opinions expressed in this article are those of the authors and do not necessarily represent the views of* ***ILSI Europe.***  Boorsma W, Snijder MB, Nijpels G, Guidone C, Favuzzi AMR, Mingrone G, Kostense, P. J. Heine, R. J**. Dekker, J. M.** Body Composition, Insulin Sensitivity, and Cardiovascular Disease Profile in Healthy Europeans. Obesity. 2008;16(12):2696-701**.**  *The RISC study was made possible by grants from the EU (QLG1CT-2001-01252), Astra-Zeneca, and Merck Sante.* ***The Dutch subcohort was supported by additional grants from*** *the Netherlands Heart foundation (2002B123) and* ***Heineken*** *NV. Further information on the RISC project and participating centers can be found on the website egir.org.*  de Rooij SR, Nijpels G, Nilsson PM, Nolan JJ, Gabriel R, Bobbioni-Harsch E, Mingrone, G**. Dekker, J. M.** Low-Grade Chronic Inflammation in the Relationship between Insulin Sensitivity and Cardiovascular Disease (RISC) Population Associations with insulin resistance and cardiometabolic risk profile. Diabetes Care. 2009;32(7):1295-301.  *The RISC study is partly supported by European Union Grant QLG1-CT-2001-01252. Additional support for the RISC study has been provided by AstraZeneca (Sweden). The EGIR Group is supported by Merck Sante (France).* ***The Dutch subcohort was supported by additional grants from*** *the Netherlands Heart Foundation (2002B123) and* ***Heineken*** *International BV*.  Pilz S, Rutters F, Nijpels G, Stehouwer CDA, Hojlund K, Nolan JJ, Balkau, B. **Dekker,** J. M. Insulin Sensitivity and Albuminuria: The RISC Study. Diabetes Care. 2014;37(6):1597-603.  *The RISC study is partly supported by European Union grant QLG1-CT-2001-01252. S.P. is supported by an EFSD Albert Renold Travel Fellowship grant. Additional support for the RISC study has been provided by AstraZeneca (Sweden). The EGIR Group is supported by Merck Sante (France).* ***The Dutch subcohort was supported by additional grants from*** *the Netherlands Heart Foundation (2002B123) and* ***Heineken*** *International BV. No other potential conflicts of interest relevant to this article were reported.* |
|  | **Other declarations of alcohol industry support** | None identified |
|  | **Declarations by co-authors** | None identified |
| Henk F. J. Hendriks | **Alcohol industry research funding declaration** | Koppes, L.L.J., Dekker, J.M., **Hendriks, H.F.J.**Bouter, L.M, Heine, R.J. Meta-analysis of the relationship between alcohol consumption and coronary heart disease and mortality in type 2 diabetic patients. Diabetologia 49, 648–652 (2006).  *The authors wish to thank I. Riphagen, Medical Library, Vrije University, Amsterdam, for her assistance with the literature search.* ***This work was supported by an unconditional grant from the Alcohol Task Force of the European branch of the*** ***International Life Sciences Institute (ILSI Europe). Industry members of this task force are Allied Domecq, Brasseries Kronenbourg, Diageo, Heineken and Moët et Chandon****. The opinions expressed in this article are those of the authors and do not necessarily represent the views of* ***ILSI Europe.***  Wiewiura JS, **Hendricks VF.** Informational pathologies and interest bubbles: Exploring the structural mobilization of knowledge, ignorance, and slack. New Media Soc. 2018;20(3):1123-38. *The author(s) disclosed receipt of the following financial support for the research, authorship and/or publication of this article:* ***This research was made possible by a grant from The Carlsberg Foundation*** *establishing the Center for Information and Bubble Studies at the University of Copenhagen.*  Schrieks IC, Wei MY, Rimm EB, Okereke OI, Kawachi I, **Hendriks HFJ,** Mukamal, K. Bidirectional associations between alcohol consumption and health-related quality of life amongst young and middle-aged women. J Intern Med. 2016;279(4):376-87.  *This work was supported by the Nurses' Health Study II grant UM1 CA176726 from the National Institutes of Health.* ***ICS and HFJH*** *were supported both by the Dutch Ministry of Economic Affairs, Agriculture and Innovation and by the* ***Dutch Foundation for Alcohol Research (SAR) representing Dutch producers of and traders in beer, wine and spirits*** *and The Netherlands Organization for Applied Scientific Research (TNO) (Grant EZ1503). Their joint aim is to independently investigate the health effects of moderate alcohol consumption. The funding sources had no role in conducting, analysing, or interpreting the study results or in the decision to submit the manuscript for publication.*  Schrieks IC, Heil ALJ, **Hendriks HFJ,** Mukamal KJ, Beulens JWJ. The Effect of Alcohol Consumption on Insulin Sensitivity and Glycemic Status: A Systematic Review and Meta-analysis of Intervention Studies. Diabetes Care. 2015;38(4):723-32.  ***I.C.S****. and* ***H.F.J.H.*** *were supported by both the Dutch Ministry of Economic Affairs, Agriculture and Innovation and the* ***Dutch Foundation for Alcohol Research****, representing Dutch producers of and traders in beer, wine, and spirits and The Netherlands Organization for Applied Scientific Research. Their joint aim is to independently study the health effects of moderate alcohol consumption.*  Beulens JWJ, de Zoete EC, Kok FJ, Schaafsma G, **Hendriks HFJ.** Effect of moderate alcohol consumption on adipokines and insulin sensitivity in lean and overweight men: a diet intervention study. Eur J Clin Nutr. 2008;62(9):1098-105.  *We acknowledge all those involved in the conduct of the study and thank the volunteers for their enthusiastic participation.* ***The research described in this article was funded by the*** ***Dutch Foundation for Alcohol Research (SAR).***  Beulens JWJ, van den Berg R, Kok FJ, Helander A, Vermunt SHF, **Hendriks HFJ.** Moderate alcohol consumption and lipoprotein-associated phospholipase A2 activity. Nutr Metab Carbiovasc Dis. 2008;18(8):539-44.  *We acknowledge all those involved in the conduct of the study and thank the volunteers for their enthusiastic participation.* ***The research described in this article was funded by the Dutch Foundation for Alcohol Research.***  Joosten MM, Balvers MGJ, Verhoeckx KCM, **Hendriks HFJ,** Witkamp RF. Plasma anandamide and other N-acylethanolamines are correlated with their corresponding free fatty acid levels under both fasting and non-fasting conditions in women. Nutr Metab. 2010;7:6.  *We gratefully acknowledge the volunteers for participation; H Fick, D Rouwendaal, A Speulman, J Jansen, I Klopping, I van den Assum, J Jacobs, E Busink, C Hoeflaken for practical work during the studies; J Catsburg, M Rondhuis, L Coulier, J Bezemer, R Ramaker and M Hekman for laboratory analyses; and E Dutman for data management;* ***These studies were partially supported by the*** ***Dutch Foundation for Alcohol Research (SAR).***  Joosten MM, de Graaf C, Rietman A, Witkamp RF, **Hendriks HFJ.** Short-term oral exposure to white wine transiently lowers serum free fatty acids. Appetite. 2010;55(1):124-9.  ***This work is partially supported by the*** ***Dutch Foundation for Alcohol Research (SAR)****.* *We gratefully acknowledge the volunteers for participation; Henriette Fick, Desiree Rouwendaal, Ineke Klopping, Angelique Speulman, Jolanda Jansen, Inge van den Assum, Jose Jacobs, Eric Busink, Christel Hoeflaken for practical work during the study: Jan Catsburg, Wouter Vaes, Marola von Lipzig, Lisette Bok, and Peter Eriksson for laboratory analyses; Ellen Dutman for data management; and Sabina Bijlsma for statistical support. MMJ designed the protocol, interpreted the data, and drafted the manuscript. MMJ and AR performed the statistical analysis. CdG and RFW provided significant advice during designing the protocol and writing of the manuscript. HFJH was involved in the design of the protocol, provided significant advice during writing of the manuscript and obtained funding. The authors have no conflict of interest with respect to the work described in the manuscript.*  Joosten MM, Schrieks IC, **Hendriks HFJ.** Effect of moderate alcohol consumption on fetuin-A levels in men and women: post-hoc analyses of three open-label randomized crossover trials. Diabetol Metab Syndr. 2014;6:5.  ***This work was supported by the Dutch Ministry of Economic Affairs, Agriculture and Innovation and by the*** ***Dutch Foundation for Alcohol Research (SAR)*** ***representing Dutch producers of and traders in beer, wine and spirits and by TNO****. Their joint aim is to independently study the health effects of moderate alcohol consumption.*  Joosten MM, van Erk MJ, Pellis L, Witkamp RF, **Hendriks HFJ.** Moderate alcohol consumption alters both leucocyte gene expression profiles and circulating proteins related to immune response and lipid metabolism in men. Br J Nutr. 2012;108(4):620-7.  ***This study was partially supported by the European Research Advisory Board (grant no. EA 08 21), the Dutch Foundation for Alcohol Research (SAR)*** *and the Dutch Ministry of Economic affairs. M. M. J. provided partial funding, designed the study, analysed and interpreted the data and wrote the manuscript. M. J. v. E. and L. P. participated in the data collection, analysed and interpreted the data and helped in drafting the manuscript. R. F. W. helped designing the study and critically reviewed the manuscript for important intellectual content. H. F. J. H. provided funding, designed the study and critically reviewed the manuscript for important intellectual content. All authors read and approved the final manuscript. We gratefully acknowledge the volunteers for participation; I. Klopping, H. Fick, D. Rouwendaal, A. Speulman, I. van den Assum, J. Jacobs, C. Hoeflaken, J. Jansen for practical work during the study; K. Toet, W. Vaes, M. von Lipzig, L. Bok and M. Rondhuis for laboratory analyses; and E. Dutman for data management. There are no conflicts of interest.*  **Joosten MM, Witkamp RF, Hendriks HFJ. Alterations in total and high-molecular-weight adiponectin after 3 weeks of moderate alcohol consumption in premenopausal women. Metab-Clin Exp. 2011;60(8):1058-63.**  ***The research described in this article was partly funded by the Dutch Foundation for Alcohol Research (SAR) and supported by a research grant from the European Research Advisory Board (ERAB)*** *grant EA 08 21. We gratefully acknowledge the volunteers for participation; H Fick, D Rouwendaal, A Speulman, J Jansen, I Klopping, I van den Assum, J Jacobs, E Busink, and C Hoeflaken for practical work during the studies; J Catsburg, W Vaes, and L Bok for laboratory analyses; E Dutman for data management; and S Bijlsma and C Rubingh for statistical support.*  Schrieks IC, Joosten MM, Klopping-Ketelaars WAA, Witkamp RF, **Hendriks HFJ.** Moderate alcohol consumption after a mental stressor attenuates the endocrine stress response. Alcohol. 2016;57:29-34.  ***This work was supported*** *both by the Dutch Ministry of Economic Affairs, Agriculture and Innovation and by the* ***Dutch Foundation for Alcohol Research (SAR****)* ***representing Dutch producers of and traders in beer, wine and spirits and TNO*** *(Grant EZ1503). Their joint aim is to independently study the health effects of moderate alcohol consumption. The authors declare there is no conflict of interest.*  Schrieks IC, Ripken D, Stafleu A, Witkamp RF, **Hendriks HFJ.** Effects of Mood Inductions by Meal Ambiance and Moderate Alcohol Consumption on Endocannabinoids and N-Acylethanolamines in Humans: A Randomized Crossover Trial. PLoS One. 2015;10(5):15.  **This work was supported both by the** Dutch Ministry of Economic Affairs, Agriculture and Innovation and by the **Dutch Foundation for Alcohol Research (SAR)** **representing Dutch producers of and traders in beer, wine and spirits** and TNO (Grant EZ1503). Their joint aim is to independently study the health effects of moderate alcohol consumption. The funders had no role in study design, data collection and analysis, decision to publish, or preparation of the manuscript  Schrieks IC, Stafleu A, Griffloen-Roose S, de Graaf C, Witkarnp RE, Boerrigter-Rijneveld R, **Hendriks, H. F. J..** Moderate alcohol consumption stimulates food intake and food reward of savoury foods. Appetite. 2015;89:77-83.  ***This work was supported both by the*** *Dutch Ministry of Economic Affairs, Agriculture and Innovation and by the* ***Dutch Foundation for Alcohol Research (SAR)*** ***representing Dutch producers of and traders in beer, wine and spirits*** *and TNO (Grant EZ1503). Their joint aim is to independently study the health effects of moderate alcohol consumption. The funding sources had no role in the study design, in conducting, analysing, or interpreting the data or in the decision to submit the article for publication.*  Schrieks IC, Stafleu A, Kallen VL, Grootjen M, Witkamp RF, **Hendriks HFJ.** The Biphasic Effects of Moderate Alcohol Consumption with a Meal on Ambiance-Induced Mood and Autonomic Nervous System Balance: A Randomized Crossover Trial. PLoS One. 2014;9(1):10.  ***This work was supported by*** *the Dutch Ministry of Economic Affairs, Agriculture and Innovation and by the* ***Dutch Foundation for Alcohol Research (SAR)*** ***representing Dutch producers of and traders in beer, wine and spirits*** *and The Netherlands Organization for Applied Scientific Research. Their joint aim is to independently study the health effects of moderate alcohol consumption. The funders had no role in study design, data collection and analysis, decision to publish, or preparation of the manuscript.*  Schrieks IC, van den Berg R, Sierksma A, Beulens JWJ, Vaes WHJ, **Hendriks HFJ.** Effect of Red Wine Consumption on Biomarkers of Oxidative Stress. Alcohol Alcohol. 2013;48(2):153-9.  ***This work was supported*** *both by the Ministry of Economic Affairs, Agriculture and Innovation and* ***by the Dutch Foundation for Alcohol Research (SAR)*** ***representing Dutch producers of and traders in beer, wine and spirits*** *and TNO. Their joint aim is to independently study the health effects of moderate alcohol consumption.*  Mukamal, K.J., Clowry, C.M., Murray, M.M., **Hendriks, H.F.,** Rimm, E.B., Sink, K.M., Adebamowo, C.A., Dragsted, L.O., Lapinski, P.S., Lazo, M. and Krystal, J.H. (2016), Moderate Alcohol Consumption and Chronic Disease: The Case for a Long‐Term Trial. Alcohol Clin Exp Res, 40: 2283-2291.  *This work was supported by grants U13AA023452, U34AA023258, and U10AA025286 from the National Institute on Alcohol Abuse and Alcoholism.* ***Drs. Hendriks and Dragsted*** ***have conducted short‐term feeding studies with alcohol at institutions (TNO, University of Copenhagen) that*** ***have received partial support from members of the*** ***alcohol industry****. Dr. Krystal has served on the advisory boards and/or holds stock in several companies with interests in psychiatric disorders.* |
|  | **Other declarations of alcohol industry support** | ***Dr Henk Hendriks spokesperson of the Scientific Committee – Beer and Health.*** *The Beer and Health initiative is supported financially by The Brewers of Europe. The realization of Birrainforma, whose management is autonomous, is made possible thanks to the external support of Assobirra https://beerandhealth.eu/scientific-committee/* |
|  | **Declarations by co-authors** | Beulens JWJ, Rimm EB, Hu FB, **Hendriks HFJ,** Mukamal KJ. Alcohol Consumption, Mediating, Biomarkers, and Risk of Type 2 Diabetes Among Middle-Aged Women. Diabetes Care. 2008;31(10):2050-5.  This work was supported by National Institutes of Health grants R01AA011181 and DK58845, a travel grant from the Dutch Heart Association, and **a research exchange award from European Research Advisory Board (J.W.J.B.).** |
| Robert J. Heine | **Alcohol industry research funding declaration** | Koppes, L.L.J., Dekker, J.M., Hendriks, H.F.J. Bouter, L.M, **Heine, R.J.** Meta-analysis of the relationship between alcohol consumption and coronary heart disease and mortality in type 2 diabetic patients. Diabetologia 49, 648–652 (2006).  *The authors wish to thank I. Riphagen, Medical Library, Vrije University, Amsterdam, for her assistance with the literature search.* ***This work was supported by an unconditional grant from the Alcohol Task Force of the European branch of the International Life Sciences Institute (ILSI Europe). Industry members of this task force are Allied Domecq, Brasseries Kronenbourg, Diageo, Heineken and Moët et Chandon****. The opinions expressed in this article are those of the authors and do not necessarily represent the views of* ***ILSI Europe****.*  Boorsma W, Snijder MB, Nijpels G, Guidone C, Favuzzi AMR, Mingrone G, Kostense**, P. J. Heine, R**. J. Dekker, J. M. Body Composition, Insulin Sensitivity, and Cardiovascular Disease Profile in Healthy Europeans. Obesity. 2008;16(12):2696-701.  *The RISC study was made possible by grants from the EU (QLG1CT-2001-01252), Astra-Zeneca, and Merck Sante.* ***The Dutch subcohort was supported by*** *additional grants from the Netherlands Heart foundation (2002B123) and* ***Heineken*** *NV. Further information on the RISC project and participating centers can be found on the website egir.org.* |
|  | **Other declarations of alcohol industry support** | None identified |
|  | **Declarations by co-authors** | None identified |
| Lex. M. Bouter | **Alcohol industry research funding declaration** | Koppes, L.L.J., Dekker, J.M., Hendriks, H.F.J.**Bouter, L.M,** Heine, R.J. Meta-analysis of the relationship between alcohol consumption and coronary heart disease and mortality in type 2 diabetic patients. Diabetologia 49, 648–652 (2006)**.**  *The authors wish to thank I. Riphagen, Medical Library, Vrije University, Amsterdam, for her assistance with the literature search.* ***This work was supported by an unconditional grant from the Alcohol Task Force of the European branch of the International Life Sciences Institute (ILSI Europe). Industry members of this task force are Allied Domecq, Brasseries Kronenbourg, Diageo, Heineken and Moët et Chandon.*** *The opinions expressed in this article are those of the authors and do not necessarily represent the views of* ***ILSI Europe****.* |
|  | **Other declarations of alcohol industry support** | None identified |
|  | **Declarations by co-authors** | None identified |
| **SUBNETWORK 26 (Contained one review – i.e. isolated subnetwork)** | | |
| J. Michael Gaziano | **Alcohol industry research funding declaration** | None identified |
|  | **Other declarations of alcohol industry support** | None identified |
|  | **Declarations by co-authors** | Petrone AB, **Gaziano JM,** Djousse L. Alcohol Consumption and Risk of Death in Male Physicians With Heart Failure. Am J Cardiol. 2014;114(7):1065-8.  ***Dr. Djousse*** *is currently serving as a Principal Investigator on an investigator-initiated research funded by GlaxoSmithKline and the California Walnut Commission, Folsom, CA. He* ***received travel reimbursement from the*** ***Wine in Moderation ASBL.*** *The authors have no other conflicts to disclose. This study was supported by grant R21HL088081 from the National Heart, Lung, and Blood Institute, Bethesda, Maryland. The PHS is supported by grants CA-34944, CA-40360, CA-097193, HL-26490, and HL-34595 from the National Institutes of Health, Bethesda, Maryland.* |
| Luc Djousse | **Alcohol industry research funding declaration** | *Barrett-Connor E, de Gaetano G,* ***Djousse L,*** *Ellison RC, Estruch R, Finkel H, Goldfinger, T. Keil, U. Lanzmann-Petithory, D. Mattivi, F. Skovenborg, E. Stockley, C. Svilaas, A. Teissedre, P. L. Thelle, D. S. Ursini, F. Waterhouse, A. L. Comments on Moderate Alcohol Consumption and Mortality. Journal of Studies on Alcohol and Drugs. 2016;77(5):834-6.*  ***Giovanni de Gaetano*** *received reimbursement for travel and other organizational costs incurred in the preparation of a consensus document on beer and health that were paid by a research grant from Epicomed Research srl, a small Italian company that had received financial support from* ***Assobirra, an Italian association of beer industrie****s.;* ***Luc Djousse has received grant funding from the Alcoholic Beverage Medical Research Foundation****.; R.* ***Curtis Ellison*** *was until 2013 (when the Institute ceased operations) the director of the Institute on Lifestyle & Health at Boston University School of Medicine, which received partial support from the* ***wine or beverage alcohol industry****. These were unrestricted donations to Boston University to support the work of the Institute in monitoring the scientific literature on alcohol and health and providing commentaries. Donors had no input into the commentaries. Unrestricted donations received between 2007 and 2013 from the* ***New York Wine & Grape Foundation, Canandaigua, NY; Diageo, Inc., New York City; and Brown-Forman Corporation, Louisville, KY.*** *In 2010,* ***Dr. Ellison*** *became a founder and Scientific Co-Director of the* ***International Scientific Forum on Alcohol Research****. Some* ***beverage industry funds*** *were used in establishing the website of this Forum (*[*www.bu.edu/alcohol-forum*](http://www.bu.edu/alcohol-forum)*). Over the past 50 years, he has been invited to give presentations at more than 100 local, regional, national, or international scientific meetings, many related to alcohol consumption, for which companies in the* ***wine industry*** *provided at least some of the support for travel expenses, including the* ***International Organisation of Vine and Wine, the Distilled Spirits Council of the United States (DISCUS), and the Washington State Grape Society****. In 2006, he helped organize an international scientific conference sponsored in part by the* ***International Center for Alcohol Policies (ICAP),*** *which at the time was funded by a consortium of major* ***alcohol producers and related organizations****.;* ***Dominique Lanzmann****-Petithory has accepted travel funds from the* ***Renaud Society*** *(an International Society of Medical Professionals with an Interest in Better Health and a Passion for Wine) to attend two scientific Meetings, supported in part by* ***wine producers.*** *She was the coordinator of the French National Research Program CANCERALCOOL, financed in part by the* ***Bordeaux Wine Inter-Professional Committee****.;* ***Erik Skovenborg*** *received reimbursement for attending the international "Health & Alcohol Symposium" in Copenhagen on November 8, 1996; the international "Women and Alcohol Symposium" in Stockholm on October 30, 1998; and the international symposium "Alcohol in moderation Health and Culture in the next millennium" in Oslo on October 27, 2000. The reimbursement was paid by GODA, a Danish organization that is funded by* ***Wine and Spirits Companies*** *and also (at that time) the* ***Brewers****. He was also a Member of the Board of Directors of* ***ERAB The European Foundation for Alcohol Research*** *from 2004 to 2016 (no fees or honoraria of any kind) and received reimbursement for attending three conferences on alcohol and health.* ***ERAB*** *and the alcohol and health conferences are sponsored by the* ***Brewers of Europe****. Reimbursement was also received for attending and speaking (no speaker's fee) at the 1st European Conference for Wine Education at the Weinakademie Osterreich/Austrian Wine Academy in Rust/Austria 2008. The* ***Austrian Wine Marketing Board*** *supported the conference.* ***Dr. Skovenborg*** *has occasionally written articles for* ***wine magazines and beer magazines*** *with comments on alcohol and health issues, for which he has received modest payments.;* ***Creina S. Stockley*** *is employed by* ***The Australian Wine Research Institute (AWRI)****, an independent, accredited scientific research institution whose board comprises industry-elected directors, the majority of whom are grape growers, viticulturists, winemakers, or wine industry consultants. The AWRI derives approximately two thirds of its funds from a statutory body, Wine Australia, which is funded from levies on tonnage for grapes that are processed into wine. Additional sources of funds have come from the Foundation for Alcohol Research and Education.; Pierre-Louis Teissedre was, until 2005, a professor at the Institut des Sciences de la Vigne et du Vin, University of Bordeaux, which received support from groups, associations, and companies in the* ***wine industry*** *to support the work of the Institute in monitoring the scientific literature on wine quality as well as health. Donors had no input into the commentaries. He has been invited to give presentations at scientific meetings related to wine and health where travel expenses were paid by the* ***International Organisation of Vine and Wine. Andrew L. Waterhouse****is a faculty member of the Department of Viticulture and Enology at the University of California since 1991. His research program has been largely supported by the* ***wine industry****, either directly from winemaking companies, associations thereof, or affiliated companies that produce products or services used in wine production.* |
|  | **Other declarations of alcohol industry support** | Petrone AB, Gaziano JM, **Djousse L.** Alcohol Consumption and Risk of Death in Male Physicians With Heart Failure. Am J Cardiol. 2014;114(7):1065-8.  ***Dr. Djousse*** *is currently serving as a Principal Investigator on an investigator-initiated research funded by GlaxoSmithKline and the California Walnut Commission, Folsom, CA. He* ***received travel reimbursement from the*** ***Wine in Moderation ASBL.*** *The authors have no other conflicts to disclose. This study was supported by grant R21HL088081 from the National Heart, Lung, and Blood Institute, Bethesda, Maryland. The PHS is supported by grants CA-34944, CA-40360, CA-097193, HL-26490, and HL-34595 from the National Institutes of Health, Bethesda, Maryland.*  ***Dr Luc Djousse was speaker at the 8th European Beer and Health Symposium.*** *The Beer and Health initiative is supported financially by The Brewers of Europe. The realization of Birrainforma, whose management is autonomous, is made possible thanks to the external support of Assobirra https://beerandhealth.eu/beer-and-health-symposium/8th-beer-and-health-symposium/speakers/dr-luc-djousse/* |
|  | **Declarations by co-authors** | None identified |
