# Supplementary material for: Alcohol, cardiovascular disease and industry funding: A co-authorship network analysis of systematic reviews
Source: Soc Sci Med. 2021 Nov;289:114450. doi: 10.1016/j.socscimed.2021.114450 (PMC8586735; doi:10.1016/j.socscimed.2021.114450)
Supplement: Multimedia component 2 [file mmc2.docx]

## Supplementary Table S2: Characteristics of Systematic Reviews for Retrieving Sample for Co-authorship Network Analysis

| **Systematic Review** | **Times Cited** | **Included Studies** | **Funding** | **Conflict of Interest** | **Individual author alcohol industry research funding declaration** | **Condition** | **Presents evidence for or against health protection** |
| --- | --- | --- | --- | --- | --- | --- | --- |
| **SUBNETWORK 1 (12 SRS)** | | | | | | | |
| Patra 2010^11^ | 195 | 26 | Global Burden of Disease (GBD) Study, NIAAA, Ontario Ministry of Health and Long Term Care. | None declared | None identified | Stroke | For |
| Rehm 2017^1^ | 22 | 25 | WHO Collaborating Centre. | SI: Queen Elizabeth II/H, David Archibald Ontario Graduate Scholarship in Science and Technology. MN PhD scholarship Rosa Luxemburg Foundation (Federal Ministry of Education and Research, Germany) and the German Academic Exchange Service (DAAD) | None identified | Cardiomyopathy | Against |
| Roerecke 2010^4^ | 192 | 17 | Global Burden of Disease (GBD) Study | None declared | None identified | CHD | Against |
| Roerecke 2011^5^ | 56 | 38 | Global Burden of Disease (GBD) Study and National Institute on Alcohol Abuse and Alcoholism | None declared | None identified | CHD | Against |
| Roerecke 2012^6^ | 141 | 43 | Global Burden of Disease Study and National Institute on Alcohol Abuse and Alcoholism | JR: scientific meetings organized or sponsored by the alcohol industry and received financial support for this participation, financial support from governments, national scientific research funds, the National Institutes of Health, the Canadian Institutes for Health Research and public health-orientated agencies in Australia, Austria, Canada, Germany and the United States and international organizations. | None identified | CHD | For |
| Roerecke 2013^2^ | 60 | 16 | Lundbeck A/S and Ontario Ministry of Health and Long-Term Care. | AG: Lundbeck and A&D Pharma, and Pfizer, JR: Lundbeck, MR: None declared | None identified | CVD | Against |
| Roerecke 2014^7^ | 123 | 10 | European Community’s Seventh Framework Programme | None declared | None identified | CHD | For |
| Roerecke 2014^8^ | 36 | 35 | European Community's Seventh Framework Programme | None declared | None identified | CHD | Against |
| Roerecke 2017^3^ | 95 | 36 | National Institute on Alcohol Abuse and Alcoholism of the National Institutes of Health | MR and JR: National Institutes of Health (NIH), National Institute on Alcohol Abuse and Alcoholism (NIAAA), JR: Lundbeck, JK, SWT, GG and OSMH: none declared | None identified | Blood pressure | Against |
| Roerecke 2018^9^ | 23 | 20 | National Institute on Alcohol Abuse and Alcoholism (NIAAA) of the National Institutes of Health | MR and JR: NIH, NIAAA, JR: Lundbeck, SWT, KJ, SLB, AV, OSMH, RJK, AOR: None declared | None identified | Hypertension | Against |
| Samokhvalov 2010^10^ | 86 | 6 | NIAAA, NIH, the Global Burden of Disease (GBD) Study and the Ontario Ministry of Health and Long Term Care. | None declared | None identified | Atrial fibrillaton | Against |
| Taylor 2009^12^ | 185 | 12 | Global Burden of Disease (GBD), BT: Canadian Institutes for Health Research | JR: scientific meetings organized or sponsored by the alcohol industry and received financial support for this participation. He has also received financial support for his research from governments, various national scientific research funds, the National Institutes of Health, the Canadian Institutes for Health Research, other public health oriented agencies in Australia, Austria, Canada, Germany, the United States | None identified | Hypertension | For |
| **SUBNETWORK 2 (6 SRS)** | | | | | | | |
| Bagnardi 2008^25^ | 118 | 6 | European Commission Public Health Project, Baseline for Monitoring Health Evolution Following Enlargement, LILT (Italian League Against Cancer), International Agency for Research on Cancer, Lyon | None declared | La Vecchia: Assobirra | CHD | For |
| Corrao 2000^26^ | 615 | 51 | MURST (Italian Ministry of the University and Scientic and Technologic Research) | Not reported | Zambon: ERAB | CHD | For |
| Costanzo 2010^22^ | 158 | 8 | European Research Advisory Board (ERAB). Dr. Vincenzo Bagnardi, University of Milan Bicocca, Milan, Italy, for providing the macro SAS. | Not reported | Costanzo: ERAB, Assobirra, International Organisation of Vine and Wine (OIV), Cervisia Consulenze. Castelnuovo: Cervisia Consulenze, ERAB, Assobirra. de Gaetano: Cervisia Consulenze, ERAB, Assobirra. Iacoviello: Cervisia Consulenze, ERAB. Donati: Cervisia Consulenze, ERAB. | CVD | For |
| Costanzo 2011^23^ | 122 | 18 | Cervisia Consulenze and Istituto Nazionale per la Comunicazione. | This was an investigator-initiated study. The partial sponsor of the study had no role in the selection of articles or conduct of the analyses or drafting of the manuscript. We disclaim any other relationships with industry that might pose a conflict of interest in connection with the submitted article. | Costanzo: ERAB, Assobirra, the Italian Association of the Beer and Malt Industries, International Organisation of Vine and Wine (OIV), Cervisia Consulenze. Castelnuovo: Cervisia Consulenze, ERAB, Assobirra. de Gaetano: Cervisia Consulenze, ERAB, Assobirra. Iacoviello: Cervisia Consulenze, ERAB. Donati: Cervisia Consulenze, ERAB. | CVD | For |
| Di Castelnuovo 2002^24^ | 388 | 26 | EU project FAIR-CT973261 and the Abruzzo Region (Programma Operativo Multiregionale “Sviluppo Locale Patti Territoriali per l’Occupazione,” Progetto 1, Sottoprogramma 9, Misura 3, Azione 3.4). | Not reported | Castelnuovo: Cervisia Consulenze, ERAB, Assobirra. de Gaetano: Cervisia Consulenze, ERAB, Assobirra. Iacoviello: Cervisia Consulenze, ERAB. Donati: Cervisia Consulenze, ERAB. | CVD | For |
| Di Castelnuovo 2006^27^ | 558 | 34 | Italian Ministry of University, Research, Education (MIUR) | None declared | Costanzo: ERAB, Assobirra, International Organisation of Vine and Wine (OIV), Cervisia Consulenze. Castelnuovo: Cervisia Consulenze, ERAB, Assobirra. de Gaetano: Cervisia Consulenze, ERAB, Assobirra. Iacoviello: Cervisia Consulenze, ERAB. Donati: Cervisia Consulenze, ERAB. | CVD | For |
| **SUBNETWORK 3 (5 SRS)** | | | | | | | |
| Brien 2011^13^ | 392 | 63 | Robert Wood Johnson Foundation project, Substance Abuse and Mental Health Services and the Administration Center for Substance Abuse Treatment. | SB: Alberta Heritage Foundation for Medical Research, PR: Canadian Institutes of Health Research, WG: Alberta Heritage Foundation for Medical Research. | Mukamal: Foundation for NIH from the alcoholic beverage industry (Anheuser-Busch InBev, Carlsberg Breweries A/S, Diageo plc, Heineken, Pernod Ricard USA LLC) | CHD | For |
| Mostofsky 2016^14^ | 62 | 23 | European Research Council, Harvard Catalyst, Harvard University and its affiliated academic healthcare centers, or the National Institutes of Health | EM: National Institutes of Health grant, Harvard Catalyst/The Harvard Clinical and National Institutes of Health, HC: Frederick Banting and Charles Best Canada Graduate Scholarship and Canadian Institutes of Health Research. | Mukamal: Foundation for NIH from the alcoholic beverage industry (Anheuser-Busch InBev, Carlsberg Breweries A/S, Diageo plc, Heineken, Pernod Ricard USA LLC) Rimm: The Europe Alcohol Task Force of the International Life Sciences Institute | CVD | For |
| Rimm 1996^16^ | 752 | 25 | International Life Sciences Institute (ILSI Europe Alcohol Task Force) | None declared | Klatsky: ABMRF Rimm and Klatsky and Grobbee and Stampfer: The Europe Alcohol Task Force of the International Life Sciences Institute | CHD | For |
| Rimm 1999^15^ | 927 | 42 | The Europe Alcohol Task Force of the International Life Sciences Institute. | EBR: Honorariums for speaking at academic conferences and travel expenses from Alcohol related organisations. | Rimm and Criqui and Stampfer and Williams and Fosher: The Europe Alcohol Task Force of the International Life Sciences Institute | CHD | For |
| Ronksley 2011^17^ | 374 | 84 | Robert Wood Johnson Foundation**,** the Substance Abuse and Mental Health Services and the Administration Center for Substance Abuse Treatment. | PER: Canadian Institutes of Health Research  SEB: Alberta Heritage Foundation for Medical Research, WAG: Canada Research Chair in Health Services Research and Alberta Heritage Foundation for Medical Research | Mukamal: Foundation for NIH from the alcoholic beverage industry (Anheuser-Busch InBev, Carlsberg Breweries A/S, Diageo plc, Heineken, Pernod Ricard USA LLC). | CVD | For |
| **SUBNETWORK 4 (4 SRS)** | | | | | | | |
| Larsson 2014^18^ | 154 | 7 | Swedish Research Council | None declared | None identified | Atrial fibrillaton | Against |
| Larsson 2015^19^ | 52 | 8 | Strategic Research Area in Epidemiology (SfoEpi) at Karolinska Institutet. | None declared | None identified | Heart failure | For |
| Larsson 2016^21^ | 55 | 29 | Swedish Stroke Association | AWo: Swedish Research Council/Committee for Research Infrastructures. SCL: Strategic Research Area in Epidemiology at Karolinska Institutet. HSM: National Institute for Health Research (NIHR), Cambridge Universities NIHR Comprehensive Biomedical Research Centre. | None identified | Stroke | For |
| Larsson 2018^20^ | 10 | 12 | Swedish Research Council | None declared | None identified | Heart failure | For |
| **SUBNETWORK 5 (3 SRS)** | | | | | | | |
| Britton 2000^28^ | 154 | 9 | UK Department for International Development. | Not reported | None identified | CHD | Against |
| Mazzaglia 2001^30^ | 70 | 41 | Not reported | Not reported | None identified | Stroke | Inconsistent |
| O'Neill 2018^29^ | 13 | 6 | UK Medical Research Council/Alcohol Research UK and the European Research Council. Other support: Cancer Research, Stroke Association, British Heart Foundation, Research Into Ageing, Academy of Medical Science. Agence Nationale De La Recherché, Agence Française de Sécurité Sanitaire de l’Environnement et du Travail, Electricité de France-Gaz de France and TGIR Cohortes Santé and US National Institutes of Health | None declared | None identified | CHD | Against |
| **SUBNETWORK 6 (2 SRS)** | | | | | | | |
| Stockwell 2016^31^ | 182 | 87 | National Institutes of Health | Not reported | None identified | CHD | For |
| Zhao 2017^32^ | 61 | 45 | U.S. National Institutes of Health | None declared | None identified | CHD | Against |
| **SUBNETWORK 7 (2 SRS)** | | | | | | | |
| Jung 2019^33^. | 1 | 11 | Korea Centers for Disease Control and Prevention | None declared | None identified | Hypertension | Against |
| Yoon 2020^34^ | 0 | 7 | Korea Centers for Disease Control and Prevention | None declared | None identified | CVD | For |
| **SUBNETWORK 8 (2 SRS)** | | | | | | | |
| Zhang 2014^35^ | 67 | 27 | The Ministry of Science and Technology of China, The Leading Talents of Science in Shanghai 2010, The Key Discipline Construction of Evidence-Based Public Health in Shanghai, The National Science Foundation of China. | None declared | None identified | Stroke | For |
| Zheng 2015^36^ | 29 | 23 | The Ministry of Science and Technology of China, The Leading Talents of Science in Shanghai 2010, The Key Discipline Construction of Evidence-Based Public Health in Shanghai, The National Science Foundation of China. | None declared | None identified | CVD | For |
| **SUBNETWORK 9 (2 SRS)** | | | | | | | |
| Reynolds 2003^37^ | 556 | 35 | National Heart, Lung, and Blood Institute. | Not reported | None identified | Stroke | For |
| Xin 2001^38^ | 445 | 15 | United States Department of Health & Human Services, National Institutes of Health (NIH) – USA, NIH National Heart Lung & Blood Institute (NHLBI) | None declared | None identified | Blood Pressure | Against |
| **ISOLATED SUBNETWORKS** | | | | | | | |
| Subnetwork 10: Barbalho 2010^39^ | 0 | 14 | Not reported | None declared | None identified | CVD | For |
| Subnetwork 11: Briasoulis 2012^40^ | 166 | 16 | Not reported | Not reported | None identified | Hypertension | For |
| Subnetwork 12: Chen 2008^41^ | 186 | 10 | LC: overseas research studentship, SJL: Higher Education Funding Council for England. GDS: MRC Centre for Causal Analyses in Translational Epidemiology. | None declared | None identified | Blood Pressure | Against |
| Subnetwork 13: Cleophas 1999^42^ | 96 | 14 | Not reported | Not reported | None identified | Myocardial Infarction | For |
| Subnetwork 14: Drogan 2012^43^ | 18 | 8 | Federal Ministry of Science, Germany, the European Union, the German Cancer Aid and the European Community | None declared | Boeing: Beer and Health Foundation, Di Guiseppe: ERAB | CVD | For |
| Subnetwork 15: Gallagher 2017^44^ | 31 | 9 | Centre for Heart Rhythm Disorders, University of Adelaide, Adelaide, Australia. CG: University of Adelaide, JMLH: National Heart Foundation of Australia and University of Adelaide. CW: National Health and Medical Research Council of Australia. MEM: University of Adelaide. MR: National Heart Foundation of Australia and National Health and Medical Research Council of Australia and University of Adelaide. DHL: University of Adelaide. PS: National Health and Medical Research Council of Australia and by the National Heart Foundation of Australia. | PS: Biosense-Webster, Medtronic, St Jude Medical, Boston Scientific, CathRx. Biotronik and Sorin | None identified | Atrial Fibrillation | Against |
| Subnetwork 16: Green 2013^45^ | 0 | 8 | BG: Leeds University Research Enterprise (LURE), MB, KB, KG: National Institute for Health Research (NIHR) & British Heart Foundation (BHF). | Nil | None identified | Abdominal Aortic Aneurysm (AAA) | For |
| Subnetwork 17: Huang 2014^46^ | 21 | 9 | National Natural Science of China | Not reported | None identified | CVD | For |
| Subnetwork 18: Huang 2017^47^ | 5 | 31 | National Natural Science Foundation of China, Natural Science Foundation of Guangdong Province and Medical and health science and technology project of Guangzhou. | None declared | None identified | Atherosclerosis | For |
| Subnetwork 19: Karpyak 2014^48^ | 25 | 24 | Not reported | Not reported | None identified | Heart Rate Variability | For |
| Subnetwork 20: Kelso 2015^49^ | 17 | 13 | Not reported | None declared | None identified | CVD | For |
| Subnetwork 21: Kodama 2011^50^ | 146 | 14 | Japan Cardiovascular Research Foundation and Ministry of Health, Labor, and Welfare, Japan. | SK and HS: Japan Society for the Promotion of Science | None identified | Atrial Fibrillation | Against |
| Subnetwork 22: Koppes 2006^51^ | 81 | 6 | Alcohol Task Force of the European branch of the International Life Sciences Institute (ILSI Europe). Industry members of this task force are Allied Domecq, Brasseries Kronenbourg, Diageo, Heineken and Moët et Chandon. | Not reported | Koppes: ILSI Europe, Heineken, Dekker: ILSI, Heineken, Hendriks: ILSI Europe, Dutch Foundation for Alcohol Research (SAR), Carlsberg, ERAB, and 'alcohol industry', Heine: ILSI Europe, Heineken. Bouter: ILSI Europe | CHD | For |
| Subnetwork 23: Lippi 2015^52^ | 10 | 16 | Not reported | None declared | None identified | Venous thromboembolism | Against |
| Subnetwork 24: McFadden 2005^53^ | 93 | 9 | Not reported | Not reported | None identified | Blood pressure | Against |
| Subnetwork 25: Naame 2019^54^ | 0 | 5 | National Natural Science Foundation of China and the Natural Science Foundation of Hunan Province | None declared | None identified | Cardiovascular risk factors in diabetics | For |
| Subnetwork 26: Padilla 2010^55^ | 16 | 6 | Not reported | None declared | Gaziano: ABMRF | Heart Failure | For |
| Subnetwork 27: Peng 2020^56^ | 0 | 28 | The Chronic Noninfectious Disease Project of National KeyR&DProgram of China', `National Natural Science Foundation of China' the `Science and Technology Planning Project of Guangdong Province, China'. | Not reported | None identified | Stroke | Inconclusive |
| Subnetwork 28: Spencer 2017^57^ | 6 | 9 | Unfunded. SMS: UK Commonwealth Scholarship Commission | None declared | None identified | Abdominal Aortic Aneurysm (AAA) | For |
| Subnetwork 29: Yang 2016^58^ | 13 | 13 | National Natural Science Foundation of China, and Science and Technological Innovation Group of Jiangsu Higher Education Institution | None declared | None identified | Coronary artery disease | For |
| Subnetwork 30: Ye 2019^59^ | 0 | 9 | Not reported | None declared | None identified | Blood pressure | For |
| Subnetwork 31: Zhang 2015^60^ | 22 | 35 | Provinces and Ministry of Education | None declared | None identified | CHD | For |

## References

1. Rehm J, Hasan OSM, Imtiaz S, et al. Quantifying the contribution of alcohol to cardiomyopathy: A systematic review. *Alcohol* 2017;61:9-15. doi: 10.1016/j.alcohol.2017.01.011

2. Roerecke M, Gual A, Rehm J. Reduction of Alcohol Consumption and Subsequent Mortality in Alcohol Use Disorders: Systematic Review and Meta-Analyses. *J Clin Psychiatry* 2013;74(12):E1181-U102. doi: 10.4088/JCP.13r08379

3. Roerecke M, Kaczorowski J, Tobe SW, et al. The effect of a reduction in alcohol consumption on blood pressure: a systematic review and meta-analysis. *Lancet Public Health* 2017;2(2):E108-E20.

4. Roerecke M, Rehm J. Irregular Heavy Drinking Occasions and Risk of Ischemic Heart Disease: A Systematic Review and Meta-Analysis. *Am J Epidemiol* 2010;171(6):633-44. doi: 10.1093/aje/kwp451

5. Roerecke M, Rehm J. Ischemic Heart Disease Mortality and Morbidity Rates in Former Drinkers: A Meta-Analysis. *Am J Epidemiol* 2011;173(3):245-58. doi: 10.1093/aje/kwq364

6. Roerecke M, Rehm J. The cardioprotective association of average alcohol consumption and ischaemic heart disease: a systematic review and meta-analysis. *Addiction* 2012;107(7):1246-60. doi: 10.1111/j.1360-0443.2012.03780.x

7. Roerecke M, Rehm J. Alcohol consumption, drinking patterns, and ischemic heart disease: a narrative review of meta-analyses and a systematic review and meta-analysis of the impact of heavy drinking occasions on risk for moderate drinkers. *BMC Med* 2014;12:11. doi: 10.1186/s12916-014-0182-6

8. Roerecke M, Rehm J. Chronic heavy drinking and ischaemic heart disease: a systematic review and meta-analysis. *Open heart* 2014;1(1):e000135. doi: 10.1136/openhrt-2014-000135

9. Roerecke M, Tobe SW, Kaczorowski J, et al. Sex-Specific Associations Between Alcohol Consumption and Incidence of Hypertension: A Systematic Review and Meta-Analysis of Cohort Studies. *J Am Heart Assoc* 2018;7(13):27. doi: 10.1161/jaha.117.008202

10. Samokhvalov AV, Irving HM, Rehm J. Alcohol consumption as a risk factor for atrial fibrillation: a systematic review and meta-analysis. *Eur J Cardiovasc Prev Rehabil* 2010;17(6):706-12. doi: 10.1097/HJR.0b013e32833a1947

11. Patra J, Taylor B, Irving H, et al. Alcohol consumption and the risk of morbidity and mortality for different stroke types--a systematic review and meta-analysis. *BMC Public Health* 2010;10:258. doi: 10.1186/1471-2458-10-258

12. Taylor B, Irving HM, Baliunas D, et al. Alcohol and hypertension: gender differences in dose-response relationships determined through systematic review and meta-analysis. *Addiction* 2009;104(12):1981-90. doi: 10.1111/j.1360-0443.2009.02694.x

13. Brien SE, Ronksley PE, Turner BJ, et al. Effect of alcohol consumption on biological markers associated with risk of coronary heart disease: systematic review and meta-analysis of interventional studies. *BMJ-British Medical Journal* 2011;342:15. doi: 10.1136/bmj.d636

14. Mostofsky E, Chahal HS, Mukamal KJ, et al. Alcohol and Immediate Risk of Cardiovascular Events A Systematic Review and Dose-Response Meta-Analysis. *Circulation* 2016;133(10):979-87. doi: 10.1161/circulationaha.115.019743

15. Rimm EB, Williams P, Fosher K, et al. Moderate alcohol intake and lower risk of coronary heart disease: meta-analysis of effects on lipids and haemostatic factors. *Br Med J* 1999;319(7224):1523-28D. doi: 10.1136/bmj.319.7224.1523

16. Rimm EB, Klatsky A, Grobbee D, et al. Review of moderate alcohol consumption and reduced risk of coronary heart disease: Is the effect due to beer, wine, or spirits? *Br Med J* 1996;312(7033):731-36.

17. Ronksley PE, Brien SE, Turner BJ, et al. Association of alcohol consumption with selected cardiovascular disease outcomes: a systematic review and meta-analysis. *BMJ-British Medical Journal* 2011;342:13. doi: 10.1136/bmj.d671

18. Larsson SC, Drca N, Wolk A. Alcohol Consumption and Risk of Atrial Fibrillation A Prospective Study and Dose-Response Meta-Analysis. *Journal of the American College of Cardiology* 2014;64(3):282-89. doi: 10.1016/j.jacc.2014.03.048

19. Larsson SC, Orsini N, Wolk A. Alcohol consumption and risk of heart failure: a dose-response meta-analysis of prospective studies. *Eur J Heart Fail* 2015;17(4):367-73. doi: 10.1002/ejhf.228

20. Larsson SC, Wallin A, Wolk A. Alcohol consumption and risk of heart failure: Meta-analysis of 13 prospective studies. *Clin Nutr* 2018;37(4):1247-51. doi: 10.1016/j.clnu.2017.05.007

21. Larsson SC, Wallin A, Wolk A, et al. Differing association of alcohol consumption with different stroke types: a systematic review and meta-analysis. *BMC Med* 2016;14(1):178. doi: 10.1186/s12916-016-0721-4

22. Costanzo S, Di Castelnuovo A, Donati MB, et al. Alcohol Consumption and Mortality in Patients With Cardiovascular Disease A Meta-Analysis. *Journal of the American College of Cardiology* 2010;55(13):1339-47. doi: 10.1016/j.jacc.2010.01.006

23. Costanzo S, Di Castelnuovo A, Donati MB, et al. Wine, beer or spirit drinking in relation to fatal and non-fatal cardiovascular events: a meta-analysis. *Eur J Epidemiol* 2011;26(11):833-50. doi: 10.1007/s10654-011-9631-0

24. Di Castelnuovo A, Rotondo S, Iacoviello L, et al. Meta-analysis of wine and beer consumption in relation to vascular risk. *Circulation* 2002;105(24):2836-44. doi: 10.1161/01.Cir.0000018653.19696.01

25. Bagnardi V, Zatonski W, Scotti L, et al. Does drinking pattern modify the effect of alcohol on the risk of coronary heart disease? Evidence from a meta-analysis. *J Epidemiol Community Health* 2008;62(7):615-19. doi: 10.1136/jech.2007.065607

26. Corrao G, Rubbiati L, Bagnardi V, et al. Alcohol and coronary heart disease: a meta-analysis. *Addiction* 2000;95(10):1505-23. doi: 10.1046/j.1360-0443.2000.951015056.x

27. Di Castelnuovo A, Costanzo S, Bagnardi V, et al. Alcohol dosing and total mortality in men and women - An updated meta-analysis of 34 prospective studies. *Archives of Internal Medicine* 2006;166(22):2437-45. doi: 10.1001/archinte.166.22.2437

28. Britton A, McKee M. The relation between alcohol and cardiovascular disease in Eastern Europe: explaining the paradox. *J Epidemiol Community Health* 2000;54(5):328-32. doi: 10.1136/jech.54.5.328

29. O'Neill D, Britton A, Hannah MK, et al. Association of longitudinal alcohol consumption trajectories with coronary heart disease: a meta-analysis of six cohort studies using individual participant data. *BMC Med* 2018;16:13. doi: 10.1186/s12916-018-1123-6

30. Mazzaglia G, Britton AR, Altmann DR, et al. Exploring the relationship between alcohol consumption and non-fatal or fatal stroke: a systematic review. *Addiction (Abingdon, England)* 2001;96(12):1743-56. doi: 10.1080/09652140120089490

31. Stockwell T, Zhao JH, Panwar S, et al. Do "Moderate" Drinkers Have Reduced Mortality Risk? A Systematic Review and Meta-Analysis of Alcohol Consumption and All-Cause Mortality. *J Stud Alcohol Drugs* 2016;77(2):185-98. doi: 10.15288/jsad.2016.77.185

32. Zhao J, Stockwell T, Roemer A, et al. Alcohol Consumption and Mortality From Coronary Heart Disease: An Updated Meta-Analysis of Cohort Studies. *J Stud Alcohol Drugs* 2017;78(3):375-86. doi: 10.15288/jsad.2017.78.375

33. Jung MH, Shin ES, Ihm SH, et al. The effect of alcohol dose on the development of hypertension in Asian and Western men: systematic review and meta-analysis. *The Korean journal of internal medicine* 2019 doi: 10.3904/kjim.2019.016

34. Yoon SJ, Jung JG, Lee SM, et al. The protective effect of alcohol consumption on the incidence of cardiovascular diseases: is it real? A systematic review and meta-analysis of studies conducted in community settings. *BMC Public Health* 2020;20(1):9. doi: 10.1186/s12889-019-7820-z

35. Zhang C, Qin YY, Chen Q, et al. Alcohol intake and risk of stroke: A dose-response meta-analysis of prospective studies. *Int J Cardiol* 2014;174(3):669-77. doi: 10.1016/j.ijcard.2014.04.225

36. Zheng YL, Lian F, Shi Q, et al. Alcohol intake and associated risk of major cardiovascular outcomes in women compared with men: a systematic review and meta-analysis of prospective observational studies. *BMC Public Health* 2015;15:11. doi: 10.1186/s12889-015-2081-y

37. Reynolds K, Lewis B, Nolen JD, et al. Alcohol consumption and risk of stroke: a meta-analysis. *JAMA* 2003;289(5):579-88.

38. Xin X, He J, Frontini MG, et al. Effects of alcohol reduction on blood pressure: a meta-analysis of randomized controlled trials. *Hypertension* 2001;38(5):1112-7. doi: 10.1161/hy1101.093424

39. Barbalho SM, Ottoboni A, Fiorini AMR, et al. Grape juice or wine: which is the best option? *Crit Rev Food Sci Nutr* 2010:14. doi: 10.1080/10408398.2019.1710692

40. Briasoulis A, Agarwal V, Messerli FH. Alcohol Consumption and the Risk of Hypertension in Men and Women: A Systematic Review and Meta-Analysis. *Journal of Clinical Hypertension* 2012;14(11):792-98. doi: 10.1111/jch.12008

41. Chen L, Davey Smith G, Harbord RM, et al. Alcohol intake and blood pressure: A systematic review implementing a Mendelian Randomization approach. *Plos Medicine* 2008;5(3) doi: 10.1371/journal.pmed.0050052

42. Cleophas TJ. Wine, beer and spirits and the risk of myocardial infarction: a systematic review. *Biomed Pharmacother* 1999;53(9):417-23. doi: 10.1016/s0753-3322(99)80121-8

43. Drogan D, Sheldrick AJ, Schutze M, et al. Alcohol Consumption, Genetic Variants in Alcohol Deydrogenases, and Risk of Cardiovascular Diseases: A Prospective Study and Meta-Analysis. *PLoS One* 2012;7(2):11. doi: 10.1371/journal.pone.0032176

44. Gallagher C, Hendriks JML, Elliott AD, et al. Alcohol and incident atrial fibrillation - A systematic review and meta-analysis. *Int J Cardiol* 2017;246:46-52. doi: 10.1016/j.ijcard.2017.05.133

45. Green B, Bailey M, Griffin K, et al. Systematic review: Alcohol consumption as a risk factor for abdominal aortic aneurysm (AAA). *Br J Surg* 2013;100:221-22.

46. Huang C, Zhan J, Liu YJ, et al. Association Between Alcohol Consumption and Risk of Cardiovascular Disease and All-Cause Mortality in Patients With Hypertension: A Meta-Analysis of Prospective Cohort Studies. *Mayo Clin Proc* 2014;89(9):1201-10. doi: 10.1016/j.mayocp.2014.05.014

47. Huang YY, Li YM, Zheng SC, et al. Moderate alcohol consumption and atherosclerosis Meta-analysis of effects on lipids and inflammation. *Wien Klin Wochen* 2017;129(21-22):835-43. doi: 10.1007/s00508-017-1235-6

48. Karpyak VM, Romanowicz M, Schmidt JE, et al. Characteristics of Heart Rate Variability in Alcohol-Dependent Subjects and Nondependent Chronic Alcohol Users. *Alcoholism (NY)* 2014;38(1):9-26. doi: 10.1111/acer.12270

49. Kelso NE, Sheps DS, Cook RL. The association between alcohol use and cardiovascular disease among people living with HIV: a systematic review. *Am J Drug Alcohol Abuse* 2015;41(6):479-88. doi: 10.3109/00952990.2015.1058812

50. Kodama S, Saito K, Tanaka S, et al. Alcohol consumption and risk of atrial fibrillation: a meta-analysis. *J Am Coll Cardiol* 2011;57(4):427-36. doi: 10.1016/j.jacc.2010.08.641 [published Online First: 2011/01/22]

51. Koppes LLJ, Dekker JM, Hendriks HFJ, et al. Meta-analysis of the relationship between alcohol consumption and coronary heart disease and mortality in type 2 diabetic patients. *Diabetologia* 2006;49(4):648-52. doi: 10.1007/s00125-005-0127-x

52. Lippi G, Mattiuzzi C, Franchini M. Alcohol consumption and venous thromboembolism: friend or foe? *Intern Emerg Med* 2015;10(8):907-13. doi: 10.1007/s11739-015-1327-0

53. McFadden CB, Brensinger CM, Berlin JA, et al. Systematic review of the effect of daily alcohol intake on blood pressure. *American journal of hypertension* 2005;18(2 Pt 1):276-86. doi: 10.1016/j.amjhyper.2004.07.020

54. Naame SA, Li DC, Huang RX. Effects of moderate red wine on cardiovascular risk factors in diabetics: a systematic review and meta-analysis of randomized controlled trials. *Toxicol Res* 2019;8(6):979-87. doi: 10.1039/c9tx00227h

55. Padilla H, Gaziano JM, Djousse L. Alcohol Consumption and Risk of Heart Failure: A Meta-Analysis. *Physician Sportsmed* 2010;38(3):84-89. doi: 10.3810/psm.2010.10.1812

56. Peng J, Wang H, Rong X, et al. Cerebral Hemorrhage and Alcohol Exposure: A Review. *Alcohol and alcoholism (Oxford, Oxfordshire)* 2020;55(1):20-27. doi: 10.1093/alcalc/agz087

57. Spencer SM, Trower AJ, Jia X, et al. Meta-analysis of the association between alcohol consumption and abdominal aortic aneurysm. *Br J Surg* 2017;104(13):1756-64. doi: 10.1002/bjs.10674

58. Yang Y, Liu DC, Wang QM, et al. Alcohol consumption and risk of coronary artery disease: A dose-response meta-analysis of prospective studies. *Nutrition* 2016;32(6):637-44. doi: 10.1016/j.nut.2015.11.013

59. Ye JH, Chen XF, Bao LG. Effects of wine on blood pressure, glucose parameters, and lipid profile in type 2 diabetes mellitus A meta-analysis of randomized interventional trials (PRISMA Compliant). *Medicine (Baltimore)* 2019;98(23):9. doi: 10.1097/md.0000000000015771

60. Zhang XY, Shu L, Si CJ, et al. Dietary Patterns, Alcohol Consumption and Risk of Coronary Heart Disease in Adults: A Meta-Analysis. *Nutrients* 2015;7(8):6582-605. doi: 10.3390/nu7085300
