# Supplementary material for: Alcohol, cardiovascular disease and industry funding: A co-authorship network analysis of systematic reviews
Source: Soc Sci Med. 2021 Nov;289:114450. doi: 10.1016/j.socscimed.2021.114450 (PMC8586735; doi:10.1016/j.socscimed.2021.114450)
Supplement: Multimedia component 3 [file mmc3.docx]

**Supplementary Figure 1: Flow Diagram of Included Studies**

1844 records retrieved with alcohol terms

270 records retrieved with additional CVD terms

179 excluded based on title/abstract

91 full-text papers ordered

31 excluded;

8 not systematic review

7 letters, editorials, meeting abstracts

6 alcohol not evaluated as risk factor

5 non-English language

3 not CVD

2 methodology papers

60 included systematic reviews
